# Ab-Initio Molecular Dynamics Simulation of Condensed-Phase Reactivity: The Electrolysis of Amino Acids and Peptides

**DOI:** 10.3390/molecules25225415

**Published:** 2020-11-19

**Authors:** Ali Kiakojouri, Ebrahim Nadimi, Irmgard Frank

**Affiliations:** 1 Theoretische Chemie, Universität Hannover, Callinstr. 3A, 30167 Hannover, Germany; kia@pci.uni-hannover.de; 2 Faculty of Electrical Engineering, K. N. Toosi University of Technology, P. O. Box 16315-1355, Tehran 1996715433, Iran; nadimi@kntu.ac.ir

**Keywords:** Car–Parrinello molecular dynamics, electrochemistry, reaction intermediates, reaction mechanisms, wastewater cleansing

## Abstract

Electrolysis is a potential candidate for a quick method of wastewater cleansing. However, it is necessary to know what compounds might be formed from bioorganic matter. We want to know if there are toxic intermediates and if it is possible to influence the product formation by the variation in initial conditions. In the present study, we use Car–Parrinello molecular dynamics to simulate the fastest reaction steps under such circumstances. We investigate the behavior of amino acids and peptides under anodic conditions. Such highly reactive situations lead to chemical reactions within picoseconds, and we can model the reaction mechanisms in full detail. The role of the electric current is to discharge charged species and, hence, to produce radicals from ions. This leads to ultra-fast radical reactions in a bulk environment, which can also be seen as redox reactions as the oxidation states change. In the case of amino acids, the educts can be zwitterionic, so we also observe complex acid–base chemistry. Hence, we obtain the full spectrum of condensed-phase chemistry.

## 1. Introduction

Novel methods of cleansing water are needed not only in arid regions on earth, but also in astronautics. Under such conditions, abundant electric energy is often available from photovoltaics. Hence, one may ask the question whether an electric current can help to decompose waste dissolved in water to nonvirulent, nontoxic substances. Electrolysis is the method of using an electric current to compose or - mostly - decompose chemical substances. Besides the well-known decomposition of water to molecular oxygen and molecular hydrogen, electrolysis is used to synthesize metals from the corresponding oxides [1]. An important example is aluminum production by the reduction of aluminum ore (Hall–Heroult process). However, the reverse reaction, namely anodizing, is also technologically important for obtaining extremely hard alumina surfaces. Another interesting application could be the reduction of, for example, carbon dioxide toward valuable substances. This concept is also known as power-to-chemicals, power-to-fuel, or power-to-gas [2].

But what does electrolysis do to bioorganic waste? We have recently investigated the electrolysis of urea and uric acid as the main contaminants in urine. For the anodic process, we found a reactivity that corresponds to the chemistry of OH^.^ radicals in aqueous solution. In the present paper, we investigate amino acids and peptides as the main constituents of bioorganic tissue.

Our approach is ab-initio molecular dynamics simulation (AIMD) or, more precisely, Car–Parrinello molecular dynamics simulation (CPMD) [3]. This method uses density functional theory (DFT) approximation [4,5,6] for a quantum mechanical description of the electrons in arbitrary molecular systems. The motion of the atoms that determines chemical reactions is simulated using Newton dynamics. It turns out that a method like DFT, which is based on the Schrodinger equation, is necessary for describing arbitrary chemical reactions. Nuclear motion, however, is very well-described using Newton’s simple concept of linear motion as long as there is no force. Car–Parrinello molecular dynamics is a way of moving the electrons together with the nuclei in a quasi-classical manner. As the electron cloud and nuclei are moved using differential equations, the approach is completely deterministic. If the same job is run two times, we obtain identical results. If, however, the initial conditions are slightly changed, we might observe completely different reactions. Obviously, we are dealing with classical chaos. CPMD is costly in terms of CPU time. Alternatives, however, are either even more costly (Born–Oppenheimer molecular dynamics, BOMD) [7] or less transferable (ReaxFF) [8,9]. There are more recent developments [10], but up to now, no method has been more important than CPMD for simulating chemical reactions [11,12,13,14,15,16]. We also cannot use cheaper methods like QM/MM, as every part of the systems investigated is potentially reactive. As an example of the ability of CPMD to find chemically reasonable structures, let us mention that we originally started with neutral amino acids in water and observed in some of the simulations the reaction toward the zwitterion, which is more stable than the neutral compound in an aqueous environment. Hence, next to radical chemistry, we have to deal with acid–base chemistry. We focus on the anodic reaction as it is mostly more interesting than the reaction at the cathode, where molecular hydrogen is formed quickly [17,18].

We are not the first ones to investigate electrolysis using AIMD. Within the Car–Parrinello community, it was mainly the work of the Sprik group and coworkers who did calculations on electrochemical problems [19,20]. Most recently, Sprik and Hutter proposed a scheme that fully includes the electrodes [21]. We are using a simpler approach that models the reactivity without direct contact to the electrodes. Our plan is to start from relatively simple systems and to understand their reactivity. Later on, we want to study more complex systems.

**Figure 1:**
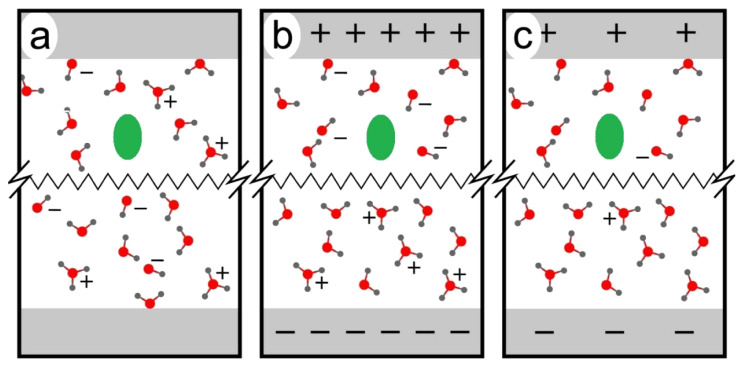
General model: We start our consideration from an aqueous solution containing a pollutant (green). (**a**) The situation before applying a potential is stable. It contains a small amount of OH^−^ and H_3_O^+^ molecules according to the ionic product (in a much smaller concentration than shown here). (**b**) If a potential is applied, there is a mass transport of the OH^−^ ions to the anode and of the H_3_O^+^ ions to the cathode. (**c**) When coming close to the electrode surfaces, the ions are discharged due to electronic tunneling. As a result, we have a pollutant in contact with radicals and with water. We take this as the starting point of our simulations.

The general idea of our approach is as follows (Figure 1): We equilibrate proteins or peptides in an aqueous environment. A Maxwell–Boltzmann distribution of velocities must be obtained to have a well-defined temperature. This happens within picoseconds. Then, we can make the system reactive by taking out electrons and protons. This simulates the anodic situation. For the cathodic situation, it would be necessary to add electrons and protons. In an electrolysis experiment, due to the voltage applied, a mass transfer of OH^−^ and H_3_O^+^ molecules takes place toward the anode and the cathode, respectively, where they are discharged. OH^.^ radicals accumulate near the anode. In silico, in order to simulate the reaction near the anode, we replace single water molecules by OH^.^ radicals. This procedure was used before (see References [17] and [18]). It is suitable for investigating processes that occur at some distance from the electrodes, that is, processes that do not need the immediate contact to the electrodes.

## 2. Results and Discussion

### 2.1. Anodic Reaction of Amino Acids

**Figure 2:**
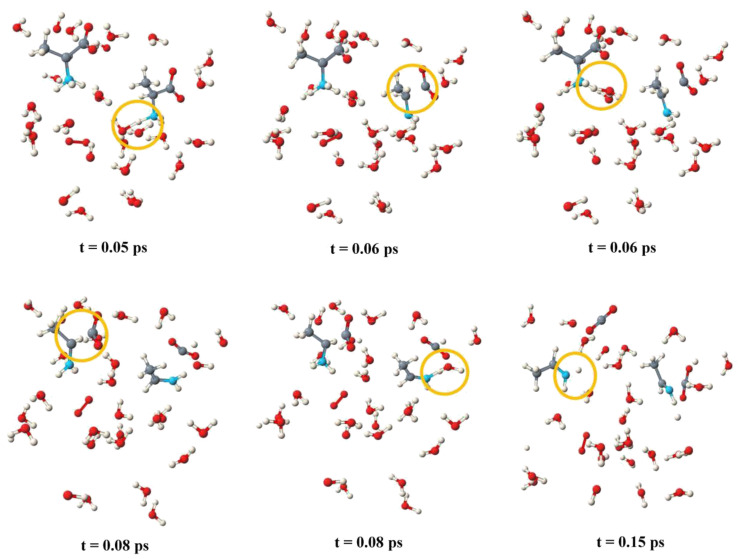
The anodic reaction of two alanine zwitterions as observed in the Car–Parrinello molecular dynamics (CPMD) simulations. The regions where reactions occur are marked with orange circles. The reaction pathway is explained in Figure 3.

Alanine is the prototype of an amino acid. In aqueous solution, alanine exists as a zwitterion. We performed simulations for both the neutral and the zwitterionic form at different initial temperatures. The initial temperatures are different from the simulation temperatures, which are not constant in a reactive simulation. According to the equipartition theorem, for an optimized system, half of the kinetic energy is converted into potential energy, leading to lower simulation temperatures. Let us focus on the simulation with two zwitterions at an initial temperature of 400 K as it exhibits the most frequent reaction pathway. The result is depicted in Figure 2. We can monitor all single reaction steps.

**Figure 3:**
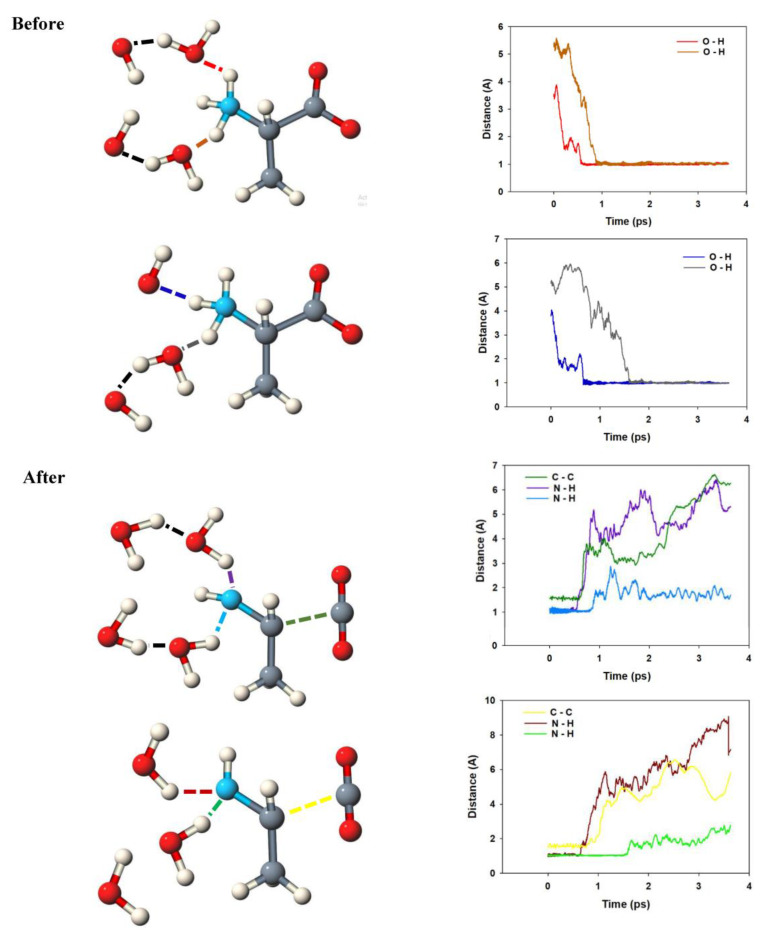
Sketch of the anodic reaction of two alanine zwitterions and behavior of the most relevant bonding interactions, deduced from the Car–Parrinello simulations.

A sketch of the molecules involved in the reaction is given in Figure 3.

The snapshots start at a point at which an oxygen molecule was already formed from OH^.^ radicals. As described before [18], we observe the formation of water wires that are similar to proton wires connected with the Grotthuss mechanism, except that our water wires involve OH^.^ radicals instead of protons and do not necessarily transport charges. The situation is complex for the zwitterion: Mediated by water molecules, two OH^.^ radicals attack at the NH_3_ group. Based on the Car–Parrinello MD simulations, we can decompose the reaction in single reaction steps and can determine what happens first. In our simulation, the first step is the formation of a hydrogen bond between a water molecule and the amino acid. This happens for both alanine zwitterions in the unit cell within about 0.2 ps (red curve and dark blue curve).

A short-lived oscillation of this hydrogen bridge is observed at 1.7–1.8 A. About 0.6 ps after the start of the reactive simulation, this distance shortens to about 1.0–1.1 A, indicating the formation of a covalent bond. At the same time, one of the N–H bonds is broken and water is formed (lilac and brown curves). Not much later, the carbon–carbon bond breaks in both simulations (green and yellow curves). The result is unstable; a few tenths of a picosecond later, the attack of a second water molecule via a water wire leads to the stable imine.

**Figure 4:**
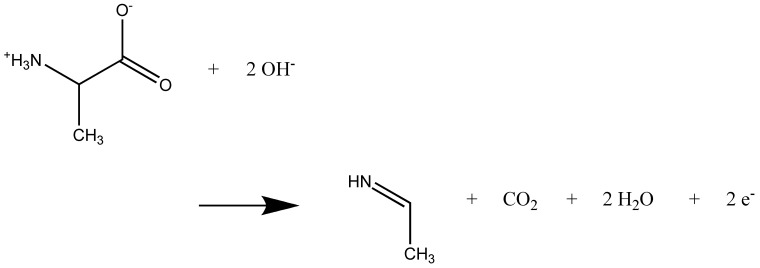
Net reaction of two alanine zwitterions as observed in the simulations.

**Figure 5:**
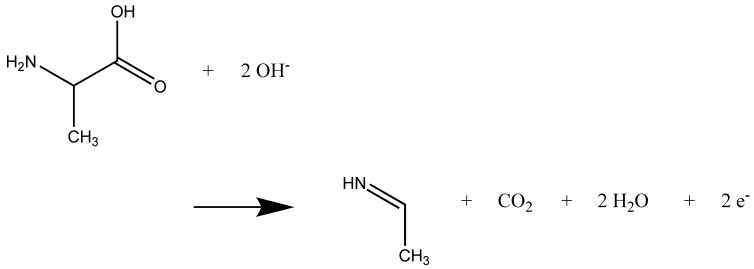
Net reaction of two alanine molecules as observed in the simulations.

The well-known net reaction that resembles the Kolbe electrolysis [22,23] is given in Figure 4. For neutral alanine, we observed a very similar reaction (Figure 5). The details for all simulations performed are given in the Supplementary Material. Among the most frequent side reactions were the formation of H_2_O_2_ and O_2_. In one case, the nitrene CH_3_-CH(N)-COOH was formed by the attack of two OH^.^ radicals at the amino group. In two cases, formation of the hemiaminal CH_3_-CH(OH)-NH_2_ was observed by attack of an OH^.^ radical at the carbon atom C after CO_2_ formation (see the Supplementary Material). For the zwitterions, the cleavage of CO_2_ is immediate after the electron transfer. For the neutral alanine molecules, the reaction has to be triggered by the attack of OH^.^ radicals at one of the hydrogen atoms of the amino groups. Note that a reaction that never occurs is the electrophilic attack of the OH^.^ radical at the nitrogen atom of the amino group. The attack at the hydrogen atoms in order to form water is way more attractive. The formation of an N–O bond is possible as a radical recombination reaction only, if several OH^.^ radicals attack.

In the Supplementary Material, a movie of the zwitterion reaction is stored. In this movie, the motion of the spin densities is shown. Red and blue are positive and negative spin densities, respectively. In the beginning, there is spin density at the triplet oxygen and at the OH^.^ radicals. In the course of the reaction, the spin density vanishes, with the only remainder at the oxygen molecule, which is now an open-shell singlet molecule, ^1^O_2_. Reaction to the more stable triplet oxygen, ^3^O_2_, would be possible by interaction with another singlet oxygen molecule.

### 2.2. Anodic Reaction of Peptides

**Figure 6:**
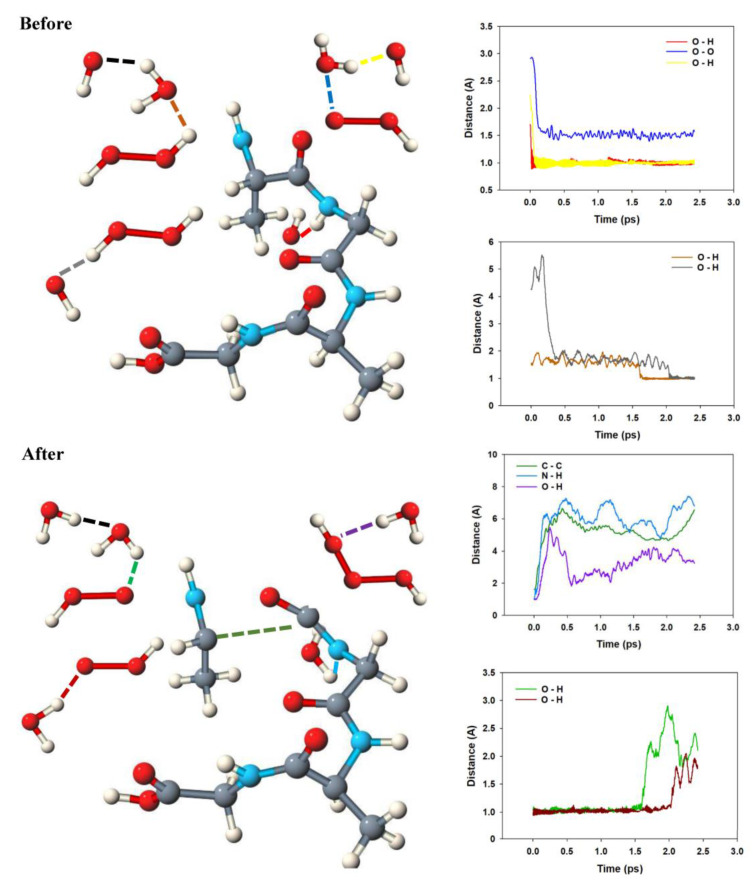
Sketch of the reaction of a small peptide and behavior of the most relevant bonding interactions. The breaking of the peptide chain is initiated by the attack of an OH^.^ radical at the hydrogen atom connected to one of the nitrogen atoms of the chain (red curve). The chain breaks at the neighboring carbon atom.

**Figure 7:**
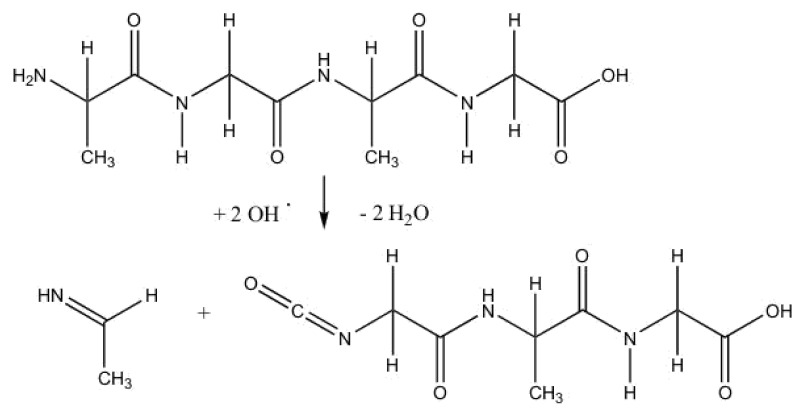
Net reaction of the peptide. The intermediary formation of OOH^.^ radicals and of the HOOOH molecule (Figure 6) can be seen as independent of the peptide reaction.

In organic materials, peptides rather than individual amino acids are found. We simulated a short peptide consisting of two glycine and two alanine moieties. The peptide seems to be more stable than the single amino acids. We observed the breaking of the chain only at high concentrations of OH^.^ radicals. As a consequence, an isocyanate group RNCO and an imine are formed (Figure 6 and Figure 7). It is known from the Bhopal catastrophe that isocyanates can be highly toxic. However, they easily hydrolyze in an aqueous environment and form relatively harmless substances like CO_2_ and R-NH_2_.

Much like the isolated amino acid, the zwitterion immediately releases a CO_2_ molecule (see the Supplementary Material).

## 3. Methods

Car–Parrinello molecular dynamics simulations using the CPMD code [3,24] have been performed using the Becke–Lee–Yang–Parr (BLYP) functional in connection with the Grimme dispersion correction [25]. The time step was chosen as 5 a.u. (0.12 fs) and the fictitious electron mass as 400 a.u. Troullier–Martins pseudopotentials as optimized for the BLYP functional were employed for describing the core electrons [26,27]. The plane-wave cutoff, which determines the size of the basis set, was set to 70.0 Rydberg. The simulation cell was 20 × 20 × 20 a.u.^3^ (10.6 ×10.6 ×10.6Å) for alanine and 25 × 25 × 25 a.u.^3^ (13.2 ×13.2 ×13.2Å^3^) for the peptide. Solutions with a density of roughly 1 g/cm^3^ were generated (2 alanine and 40 water molecules or one alanine-glycine-alanine-glycine system plus 62 water molecules). After equilibration of stable, neutral, closed-shell systems, reactive species were generated by removing four protons and four electrons, leading to OH^.^ radicals. For the reactive simulations, the spin-unrestricted version of Kohn–Sham theory was employed [28]. Typically, data were accumulated for 3 ps, before more reactive species were added in order to continue the reaction. Total simulation times were on the order of 10 ps. We also performed calculations using Nose thermostats and with Born–Oppenheimer molecular dynamics. While the conservation of classical energy looks better than that with CPMD, this did not result in a different reactivity. CPMD simulations with a smaller time step (2 a.u.) and a smaller fictitious electron mass (200 a.u.) showed a slightly better behavior concerning the energies, while the overall reactivity stayed the same (see the Supplementary Material). This observation is attributed to the use of the spin-unrestricted version of Kohn–Sham theory, which tends to be less stable than the closed-shell version, but is needed when describing radicals.

## 4. Conclusions

To conclude, in our simulations of alanine under anodic conditions, we observe mainly the decomposition to carbon dioxide, ethanimine, and water. This is clearly the predominant reaction and may be observed also for other amino acids. Ethanimine is a reactive intermediate which, in higher concentrations, easily polymerizes. It is stable on the time scale of our simulations. The reactions are mediated by water chains. This reactivity is characteristic of the bulk environment and can be observed only if the whole electronic system is treated at a quantum mechanical level.

As side reactions, we were able to observe the reaction to a nitrene and to a hemiaminal as unstable intermediates occurring at high OH^.^ concentrations. Molecular oxygen was also formed, as well as all the intermediates leading to molecular oxygen, that is, the OOH^.^ radical and the H_2_O_2_ molecule. Peptides show a similar but lower reactivity, except that we observe breaking of the chain at high OH concentrations. Under these conditions, HOOOH, known as trioxidane, was also observed, which in the experiment, easily decomposes in an aqueous environment, but is stable on the time scale of our simulation.

As the reactions are spontaneous after electron transfer, we can simply observe the full reaction. Our movies show the continuous change in the electronic structure during the reaction, which emphasizes the rigor of our approach. We can show not only how the nuclei moves from the educt to the product state, but also that the electronic cloud moves smoothly from one state to the other.

The disadvantages and limitations of our approach should also be mentioned: We observe reactions that are only completed within a few picoseconds. We can trigger reactions by changing the charge status of a system; however, we fail to observe reactions that need a certain arrangement of the reaction partners, that is, reactions for which entropy matters. On the other hand, our concentration of OH^.^ radicals is too high compared to the experiment; hence, we overestimate reactions that need the simultaneous approach of two radicals. However, such reactions become more important on longer timescales, as water wires can be formed. This compensates to some degree the effect of too high a concentration of radicals.

There are many more reaction setups that could be interesting in the context of ultrafast processes under electrolytic conditions. Let us just mention the formation of amino acids from simpler substances, which was reported earlier using contact glow discharge electrolysis (CGDE) [29,30]; for a review, see Reference [31]. Molecular dynamics simulations can add a microscopic explanation of complex reaction schemes by showing every single reaction step. An understanding of the full reaction mechanism may help to obtain the desired products in higher yields.

## Supplementary Materials

The following are available online, frank.suppl.pdf: main supplementary file, alanine.mpg: movie of one of the simulation runs, in1: sample input file.

## References

[1] Holleman A.F., Wiberg E. (1749). Lehrbuch der Anorganischen Chemie.

[2] Sternberg A., Bardow A. (2015). Power-to-What?–Environmental assessment of energy storage systems. Energy Environ. Sci..

[3] Car R., Parrinello M. (1985). Unified Approach for Molecular Dynamics and Density-Functional Theory. Phys. Rev. Lett..

[4] Hohenberg P., Kohn W. (1964). Inhomogeneous electron gas. Phys. Rev..

[5] Kohn W., Sham L.J. (1965). Self-consistent equations including exchange and correlation effects. Phys. Rev..

[6] Becke A.D. (1988). Density-functional exchange-energy approximation with correct asymptotic behavior. Phys. Rev..

[7] Marx D., Hutter J., Grotendorst J. (2000). Modern Methods and Algorithms of Quantum Chemistry. Ab Initio Molecular Dynamics: Theory and Implementation.

[8] Van Duin A.C.T., Dasgupta S., Lorant F., Iii W.A.G. (2001). ReaxFF: A Reactive Force Field for Hydrocarbons. J. Phys. Chem..

[9] Chenoweth K., Van Duin A.C.T., Goddard W.A. (2008). ReaxFF Reactive Force Field for Molecular Dynamics Simulations of Hydrocarbon Oxidation. J. Phys. Chem. A.

[10] Alonso J.L., Andrade X., Echenique P., Falceto F., Prada-Gracia D., Rubio A. (2008). Efficient formalism for large-scale ab initio molecular dynamics based on time-dependent density funtional theory. Chem. Phys. Lett..

[11] Parrinello M. (1997). From silicon to RNA: The coming of age of ab initio molecular dynamics. Solid State Commun..

[12] Fois E., Gamba A., Tabacchi G. (2000). First-principles simulation of the intracage oxidation of nitrite to nitrate sodalite. Chem. Phys. Lett..

[13] Boero M., Parrinello M., Terakura K. (1998). First Principles Molecular Dynamics Study of Ziegler−Natta Heterogeneous Catalysis. J. Am. Chem. Soc..

[14] Frank I., Parrinello M., Klamt A. (1998). Insight into Chemical Reactions from First-Principles Simulations: The Mechanism of the Gas-Phase Reaction of OH Radicals with Ketones. J. Phys. Chem..

[15] Reinhardt S., Marian C., Frank I. (2001). The influence of excess ammonia on the mechanism of the reaction of boron trichloride with ammonia–An ab initio molecular dynamics study. Angew. Chem..

[16] Nonnenberg C., Frank I. (2008). Formation and decay of tetrazane derivates–a Car-Parrinello molecular dynamics study. Phys. Chem. Chem. Phys..

[17] Hofbauer F., Frank I. (2011). Electrolysis of Water in the Diffusion Layer: First-Principles Molecular Dynamics Simulation. Chem. A Eur. J..

[18] Frank I. (2019). Ab-Initio Molecular Dynamics Simulation of the Electrolysis of Waste Water. ChemistrySelect.

[19] Blumberger J., Bernasconi L., Tavernelli I., Vuilleumier R., Sprik M. (2004). Electronic structure and solvation of copper and silver ions: A theoretical picture of a model aqueous redox reaction. J. Am. Chem. Soc..

[20] Blumberger J., Tateyama Y., Sprik M. (2005). Ab initio molecular dynamics simulation of redox reactions in solution. Comput. Phys. Commun..

[21] Zhang C., Sayer T., Hutter J., Sprik M. (2020). Modelling electrochemical systems with finite field molecular dynamics. J. Phys. Energy.

[22] Tomilov A.P., Fioshin M.Y. (1963). Free radical reactions in the electrolysis of organic compounds. Russ. Chem. Rev..

[23] Wiebe A., Gieshoff T., Möhle S., Rodrigo E., Zirbes M., Waldvogel S.R. (2018). Electrifying organic synthesis. Angew. Chem. Int. Ed..

[24] CPMD Copyright IBM Corp, 1990–2008; Copyright MPI für Festkörperforshung Stuttgart 1997–2001. http://www.cpmd.org/.

[25] Grimme S. (2006). Semiempirical GGA-type density functional constructed with a long-range dispersion correction. J. Comput. Chem..

[26] Troullier N., Martins J.L. (1991). Efficient pseudopotentials for plane-wave calculations. Phys. Rev. B.

[27] Boero M., Parrinello M., Terakura K., Weiss H. (2002). Car—Parrinello study of Ziegler—Natta heterogeneous catalysis: Stability and destabilization problems of the active site models. Mol. Phys..

[28] Gunnarsson O., Lundqvist B.I. (1976). Exchange and correlation in atoms, molecules, and solids by the spin-density-functional formalism. Phys. Rev. B.

[29] Harada K., Suzuki S., Harada S.S.K. (1977). Formation of amino acids from elemental carbon by contact glow discharge electrolysis. Nat. Cell Biol..

[30] Harada K., Nomoto M.M., Gunji H. (1981). Formation of amino acids from aliphatic amines by contact glow discharge electrolysis. Tetrahedron Lett..

[31] Gupta S.K.S. (2017). Contact Glow Discharge Electrolysis: A Novel Tool for Manifold Applications. Plasma Chem. Plasma Process..

