# Supplementary material for: Ab-Initio Molecular Dynamics Simulation of Condensed-Phase Reactivity: The Electrolysis of Amino Acids and Peptides
Source: Molecules. 2020 Nov 19;25(22):5415. doi: 10.3390/molecules25225415 (PMC7699423; doi:10.3390/molecules25225415)
Supplement: Supplementary file 1 [file molecules-25-05415-s001.zip › frank/frank.suppl.pdf]

Supplementary Material

Method

Car-Parrinello molecular dynamics simulations [1–3] have been performed using the Becke-Lee-Yang-Parr (BLYP) functional in connection with the Grimme dispersion correction [4]. The time step was chosen as 5 a.u. (0.12 fs) and the fictitious electron mass as 400 a.u. Troullier-Martins pseudopotentials as optimized for the BLYP functional were employed for describing the core electrons [5, 6]. The plane-wave cutoff which determines the size of the basis set, was set to 70.0 Rydberg. The simulation cell size was 20 x 20 x 20 $a.u.^3$ (10.6 x 10.6 x 10.6 $\AA^3$). Solutions with a density of roughly 1 g/cm^3 were generated. After equilibration of stable, neutral closed-shell systems, reactive species were generated by removing four protons and four electrons, leading to $OH^{\cdot}$ radicals. For the reactive simulations, the spin-unrestricted version of Kohn-Sham theory was employed [7]. Typically, data were accumulated for 3 ps, before more reactive species were added in order to continue the reaction. Total simulation times were in the order of 10 ps. We also performed calculations using Nose thermostats and with Born-Oppenheimer molecular dynamics. While the energy conservation looks better than with CPMD, this did not result in a different reactivity.

[1] R. Car and M. Parrinello, Phys. Rev. Lett. **55**, 2471 (1985).

[2] D. Marx and J. Hutter, *Ab Initio Molecular Dynamics: Basic Theory and Advanced Methods* (Cambridge University Press, Cambridge, 2009).

[3] CPMD, Version 4.1, J. Hutter et al., http://www.cpmd.org/, Copyright IBM Corp 1990-2015, Copyright MPI für Festkörperforschung Stuttgart 1997-2001.

[4] S. Grimme, J. Comput. Chem. **27**, 1787 (2006).

[5] N. Troullier and J. L. Martins, Phys. Rev. **43**, 1993 (1991).

[6] M. Boero, M. Parrinello, K. Terakura, and H. Weiss, Mol. Phys. **100**, 2935 (2002).

[7] O. Gunnarsson and B. I. Lundqvist, Phys. Rev. B **13**, 4274 (1976).

Deposited as separate file:

alanine.mpg: Movie of the reaction of two zwitterions with eight OH radicals at 400 K.

in1: Input file for CPMD version 4.1.

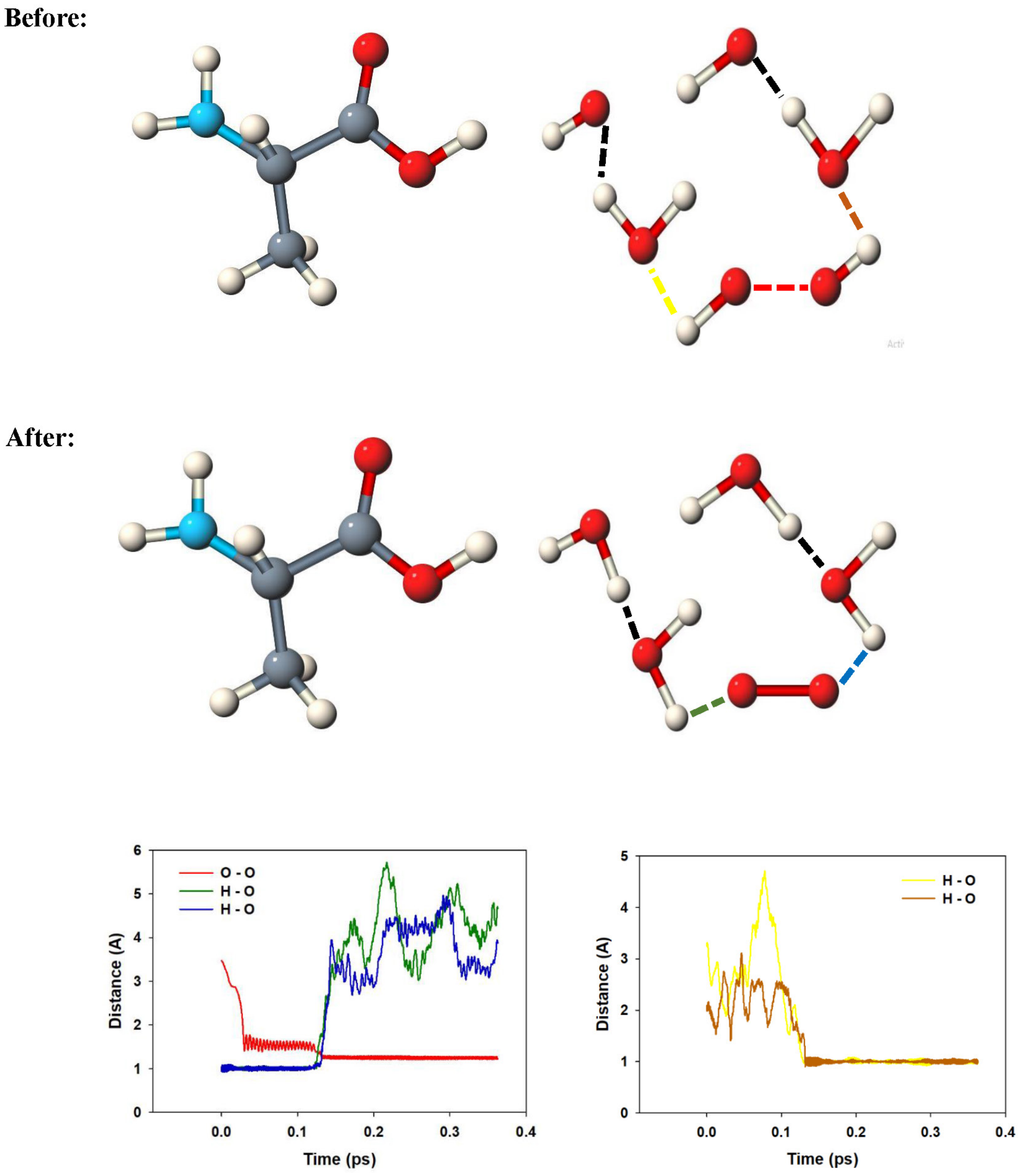

FIG. 1. Two alanine molecules in solution with four OH radicals. The initial temperature is 300K.

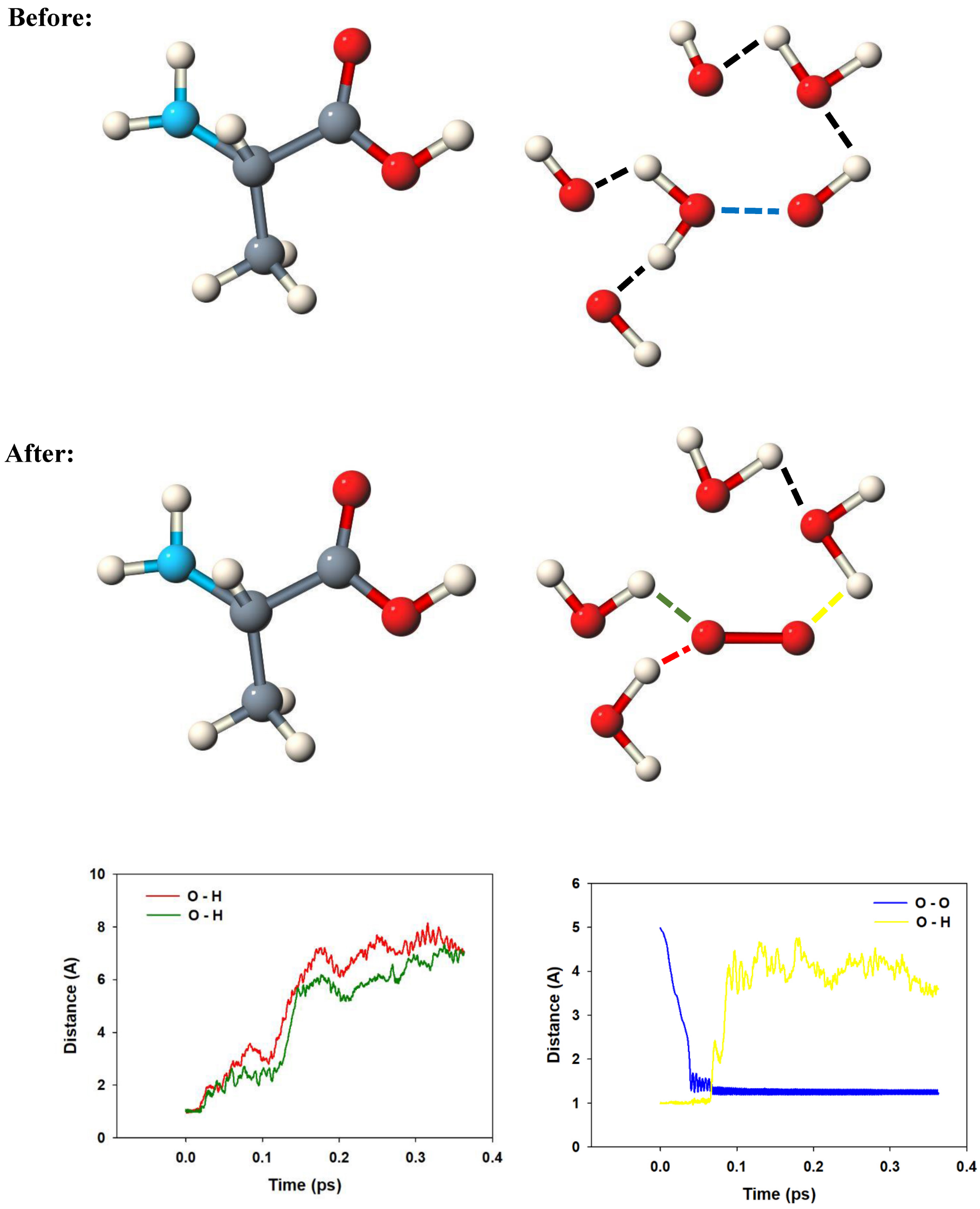

FIG. 2. Two alanine molecules in solution with eight OH radicals. The initial temperature is 300K.

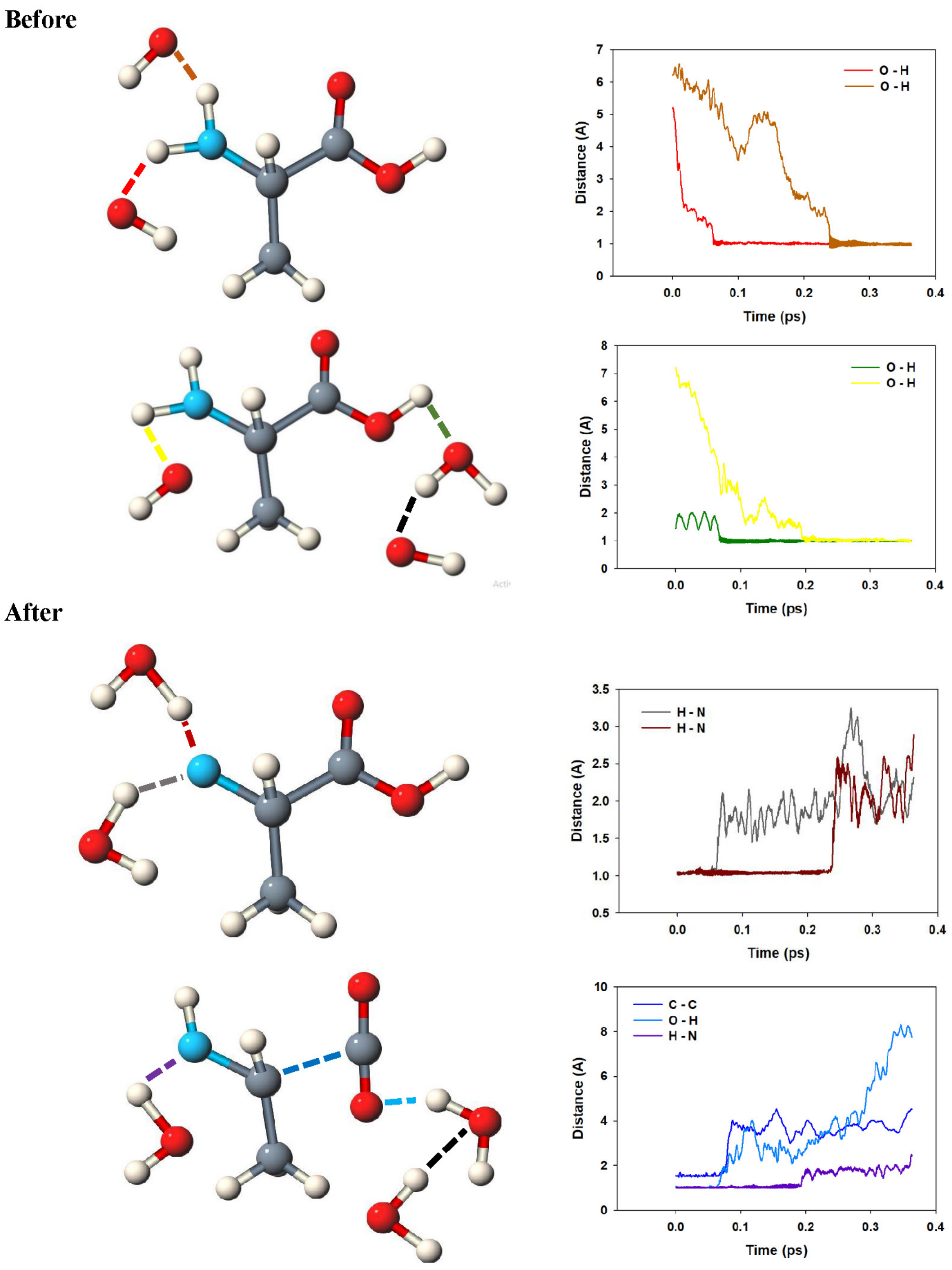

FIG. 3. Two alanine molecules in solution with eight OH radicals. The initial temperature is 400K.

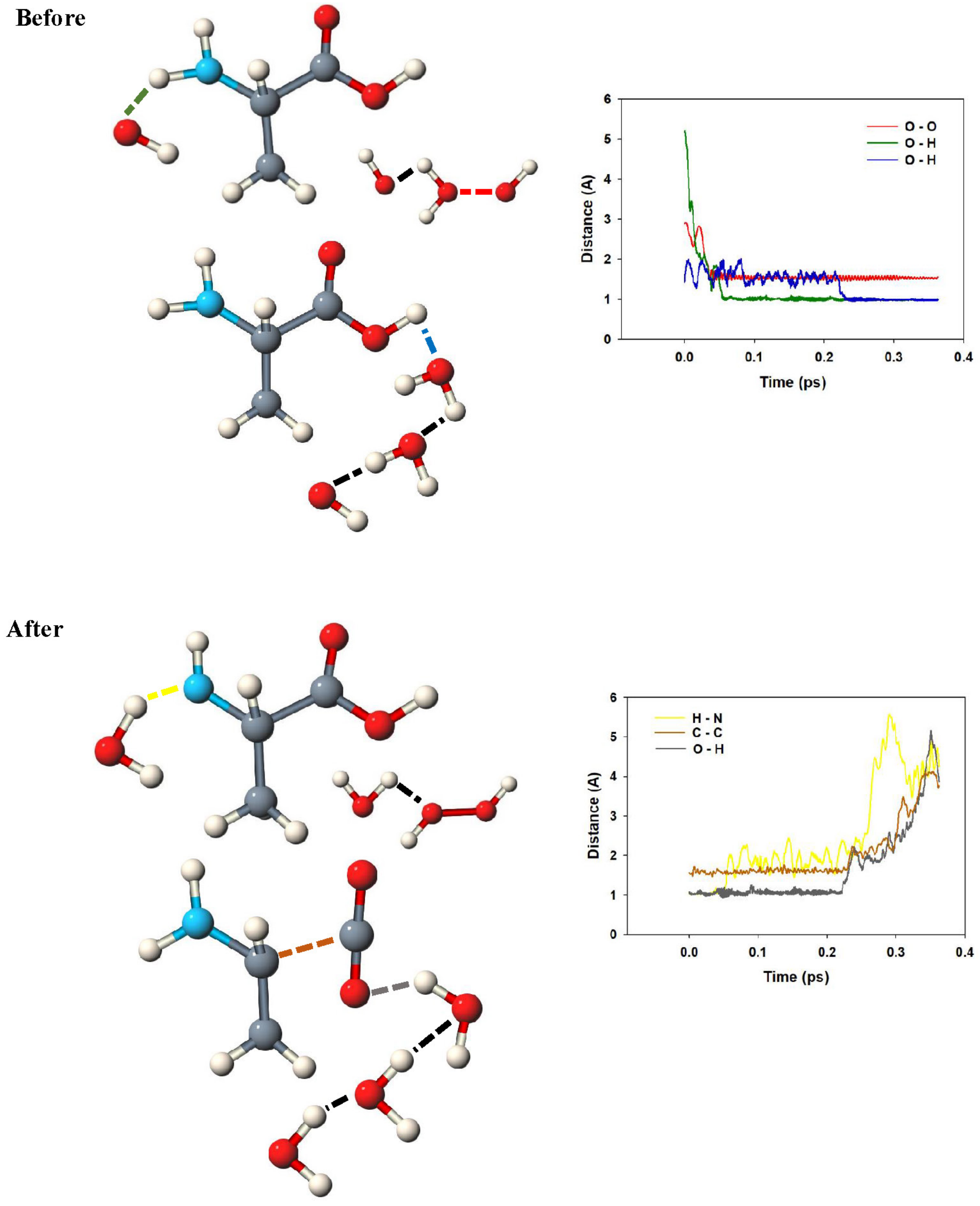

FIG. 4. Two alanine molecules in solution with eight OH radicals. The initial temperature is 500K.

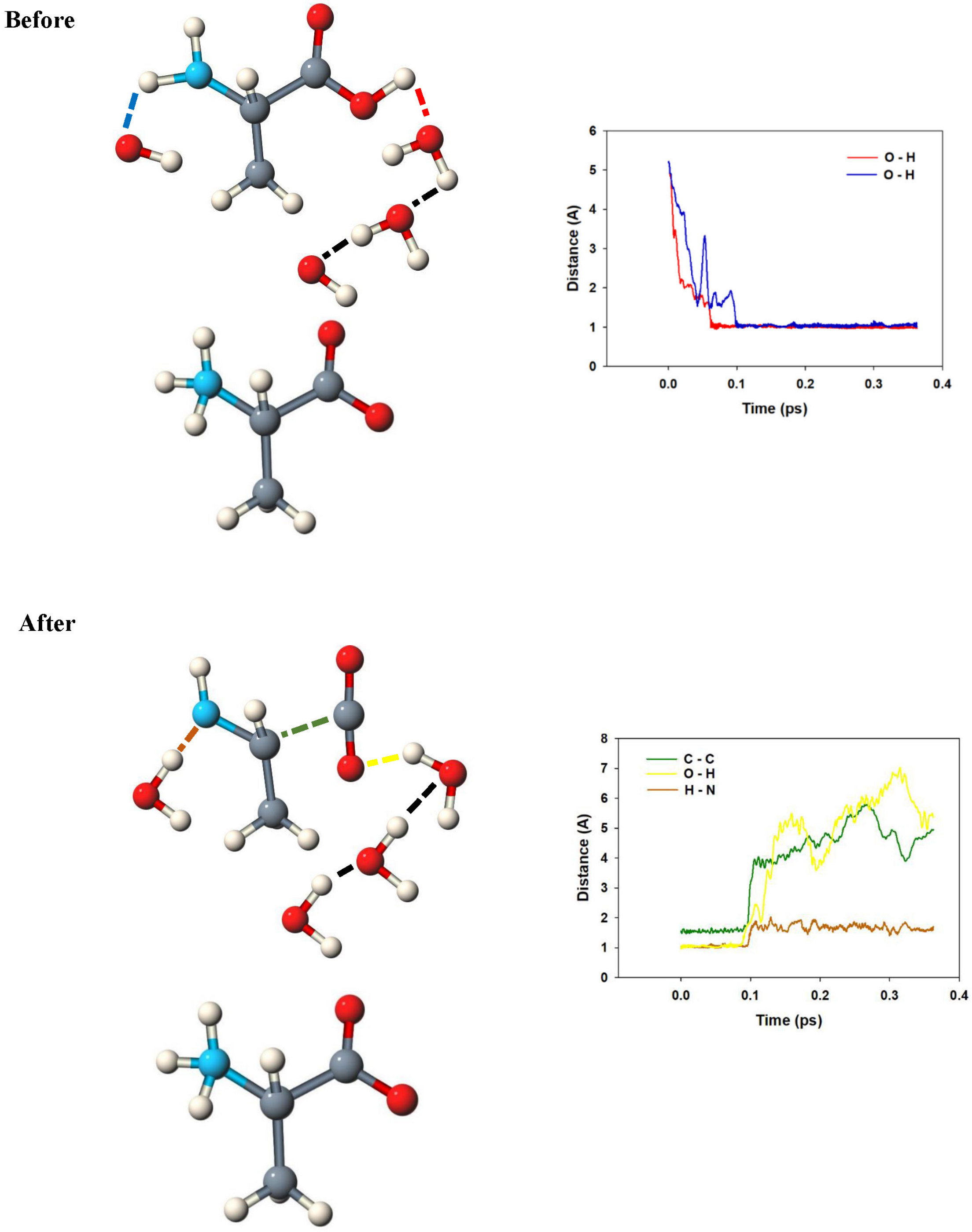

FIG. 5. One alanine molecule and one zwitterion in solution with eight OH radicals. The initial temperature is 300K.

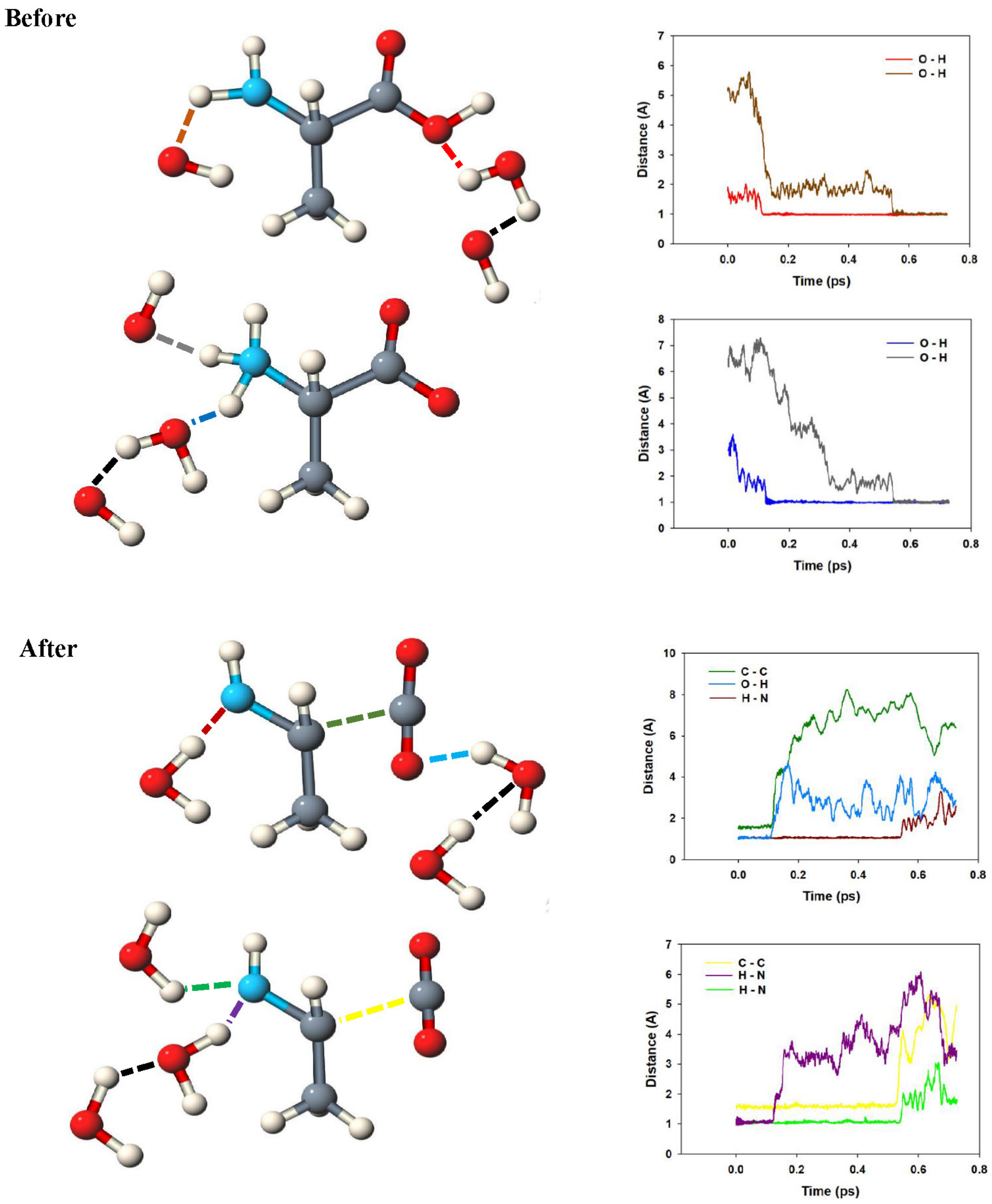

FIG. 6. One alanine molecule and one zwitterion in solution with eight OH radicals. The initial temperature is 400K.

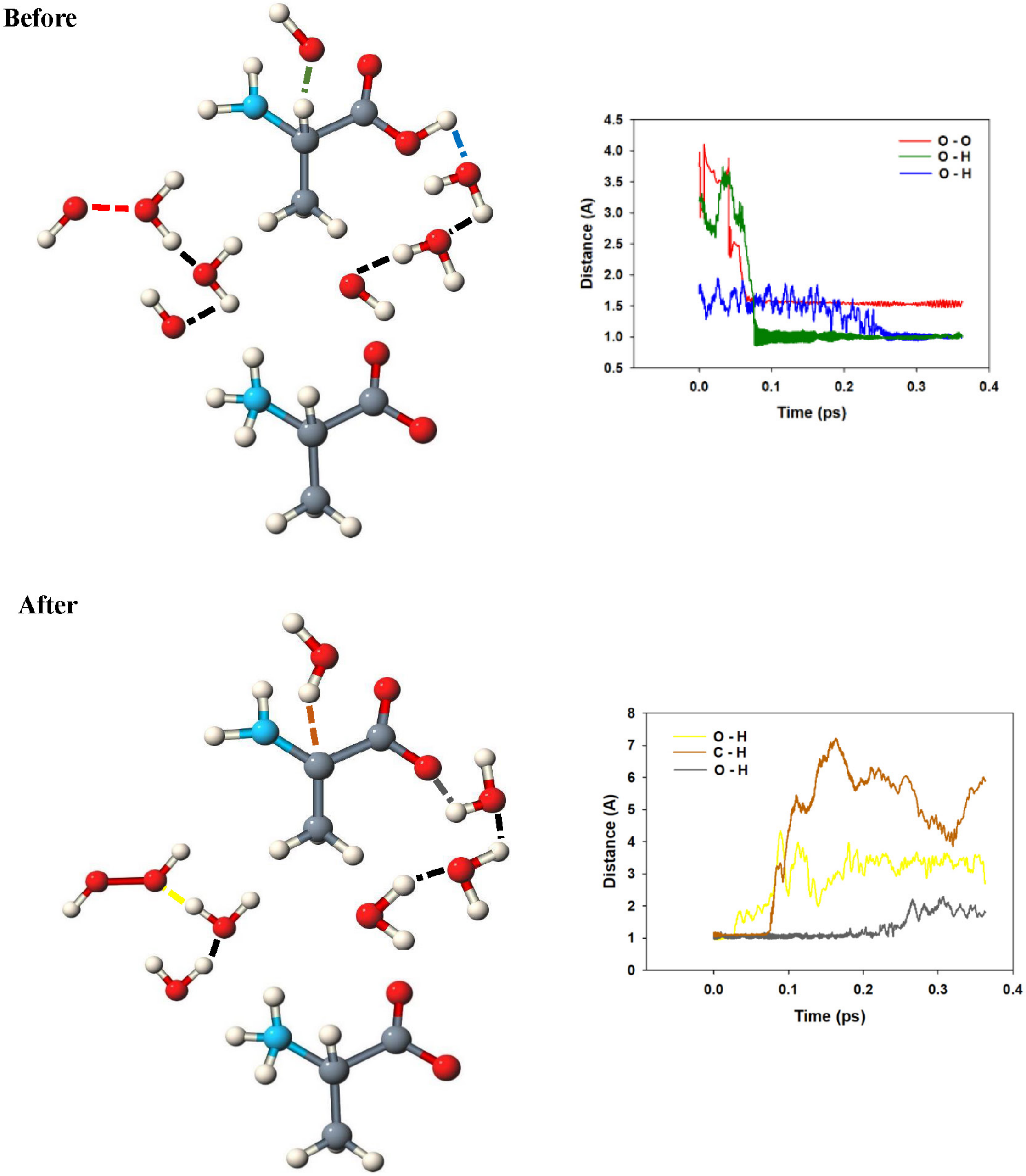

FIG. 7. One alanine molecule and one zwitterion in solution with eight OH radicals. The initial temperature is 500K.

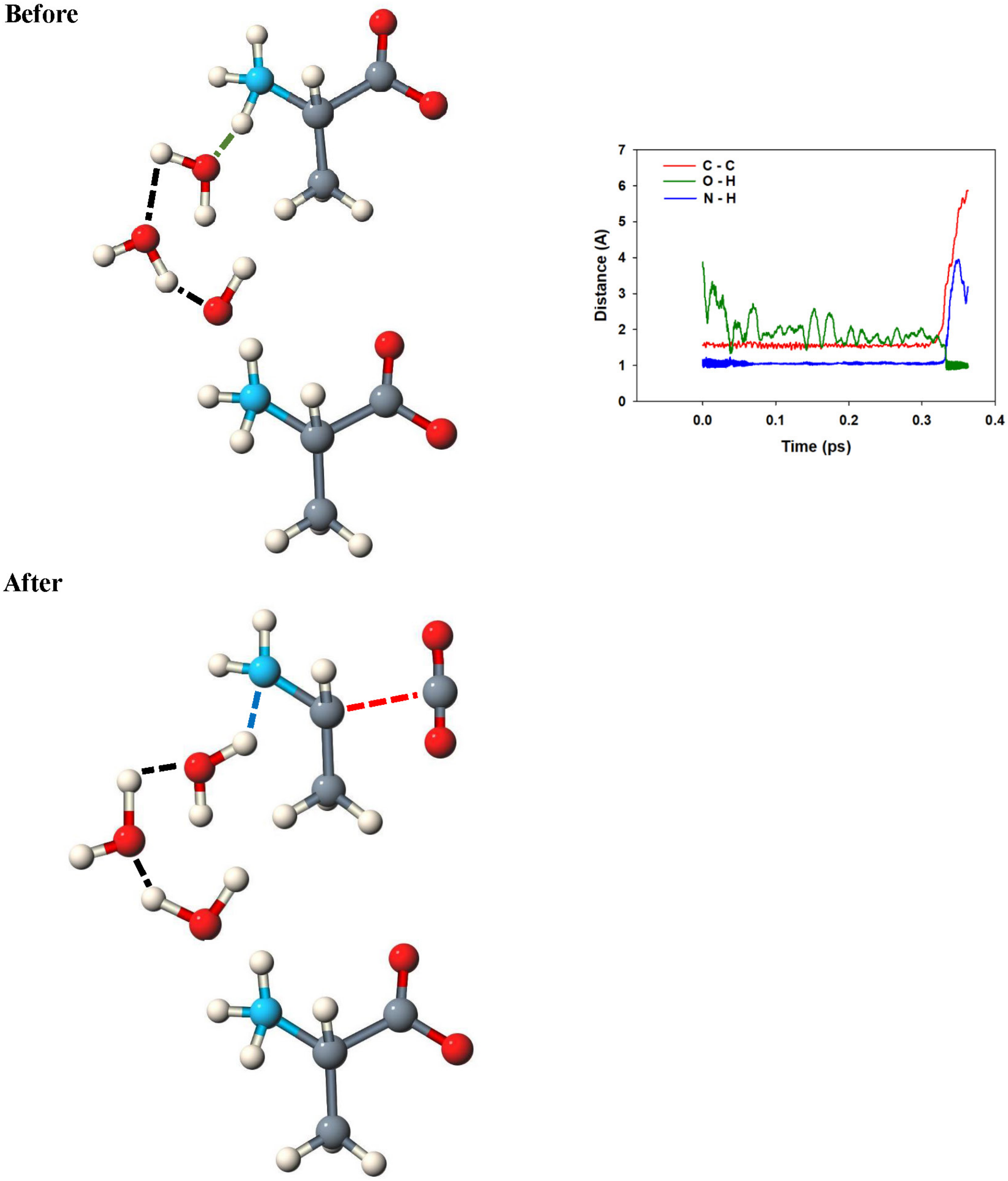

FIG. 8. Two zwitterions in solution with eight OH radicals. The initial temperature is 300K.

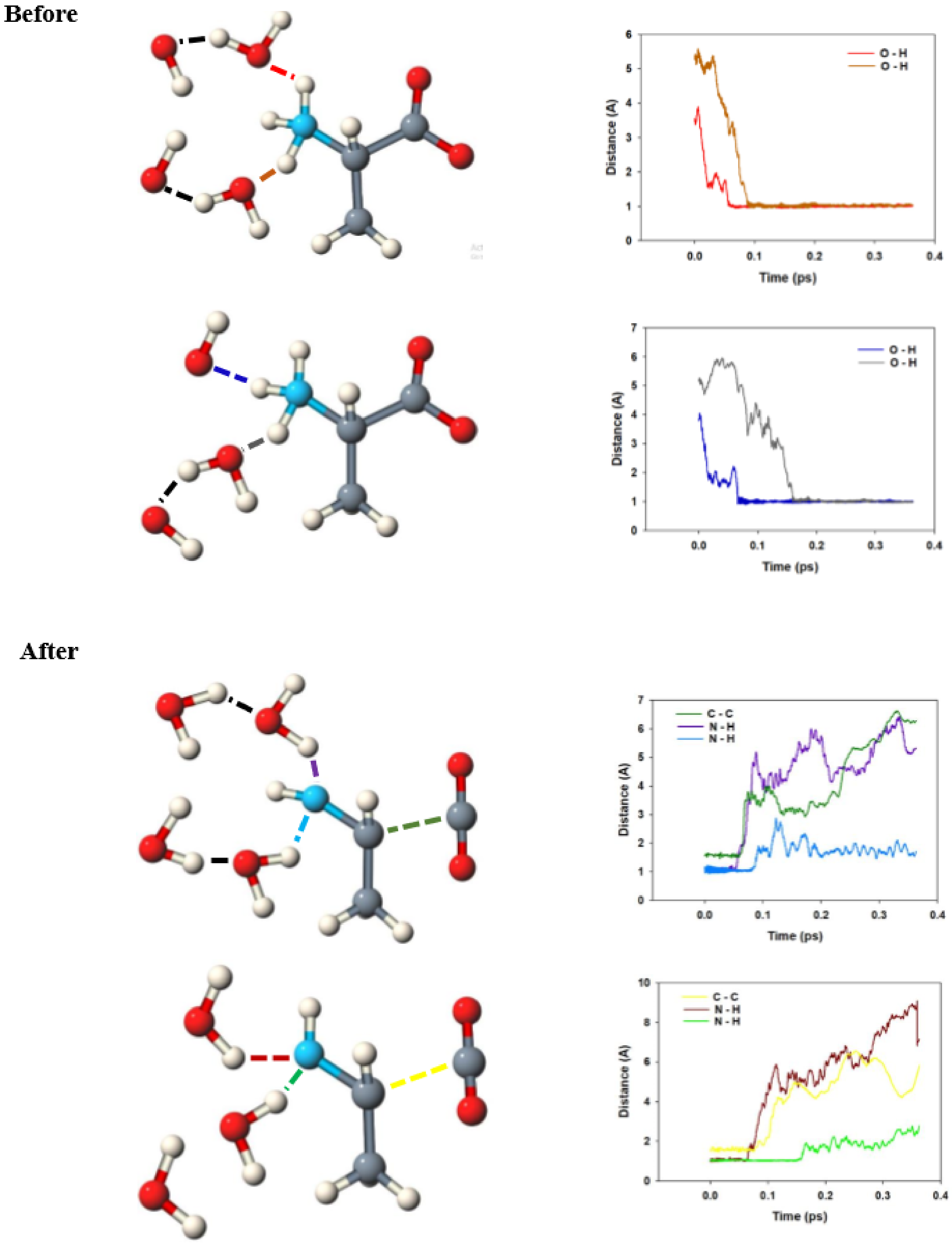

FIG. 9. Two zwitterions in solution with eight OH radicals. The initial temperature is 400K.

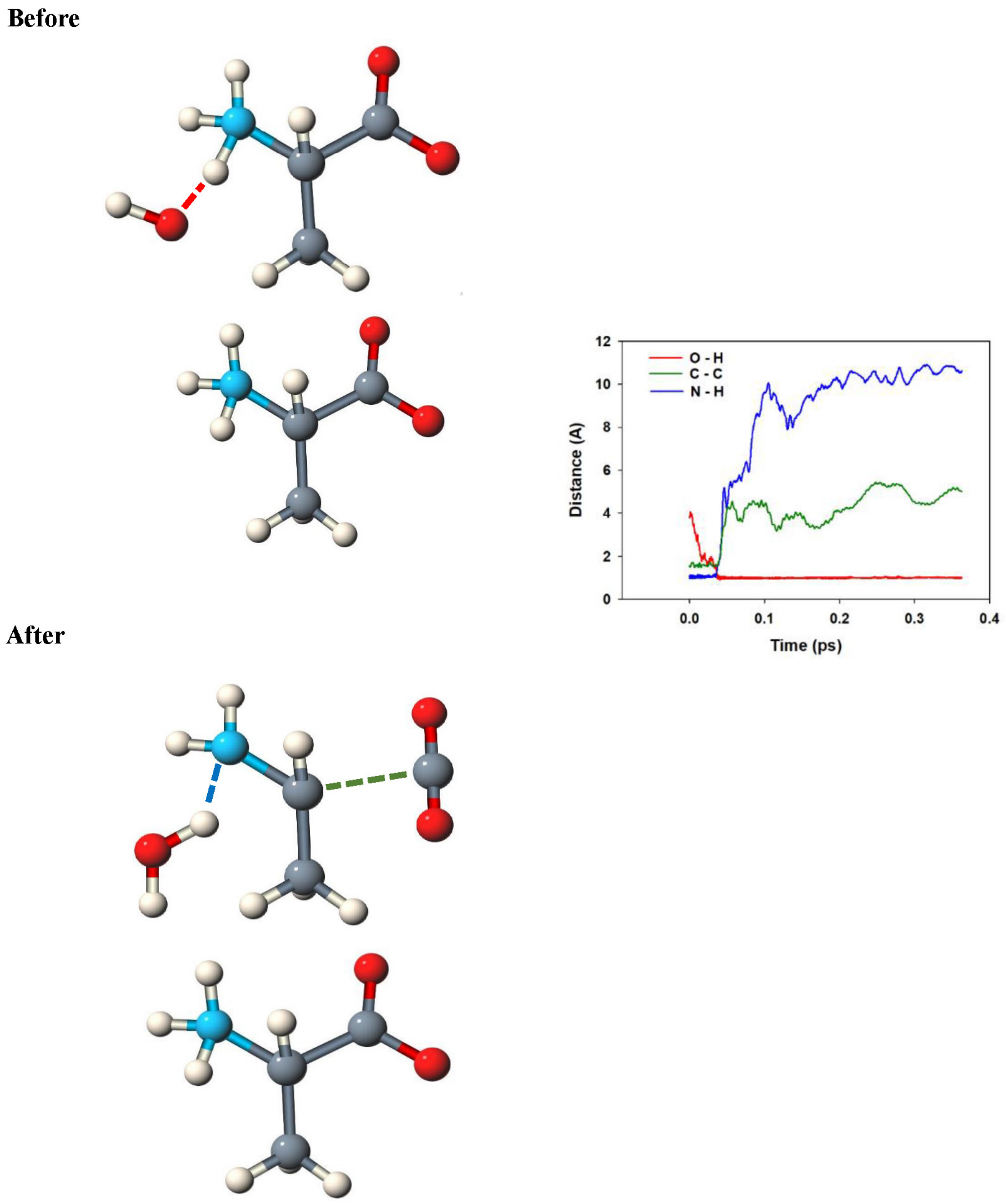

FIG. 10. Two zwitterions in solution with eight OH radicals. The initial temperature is 500K.

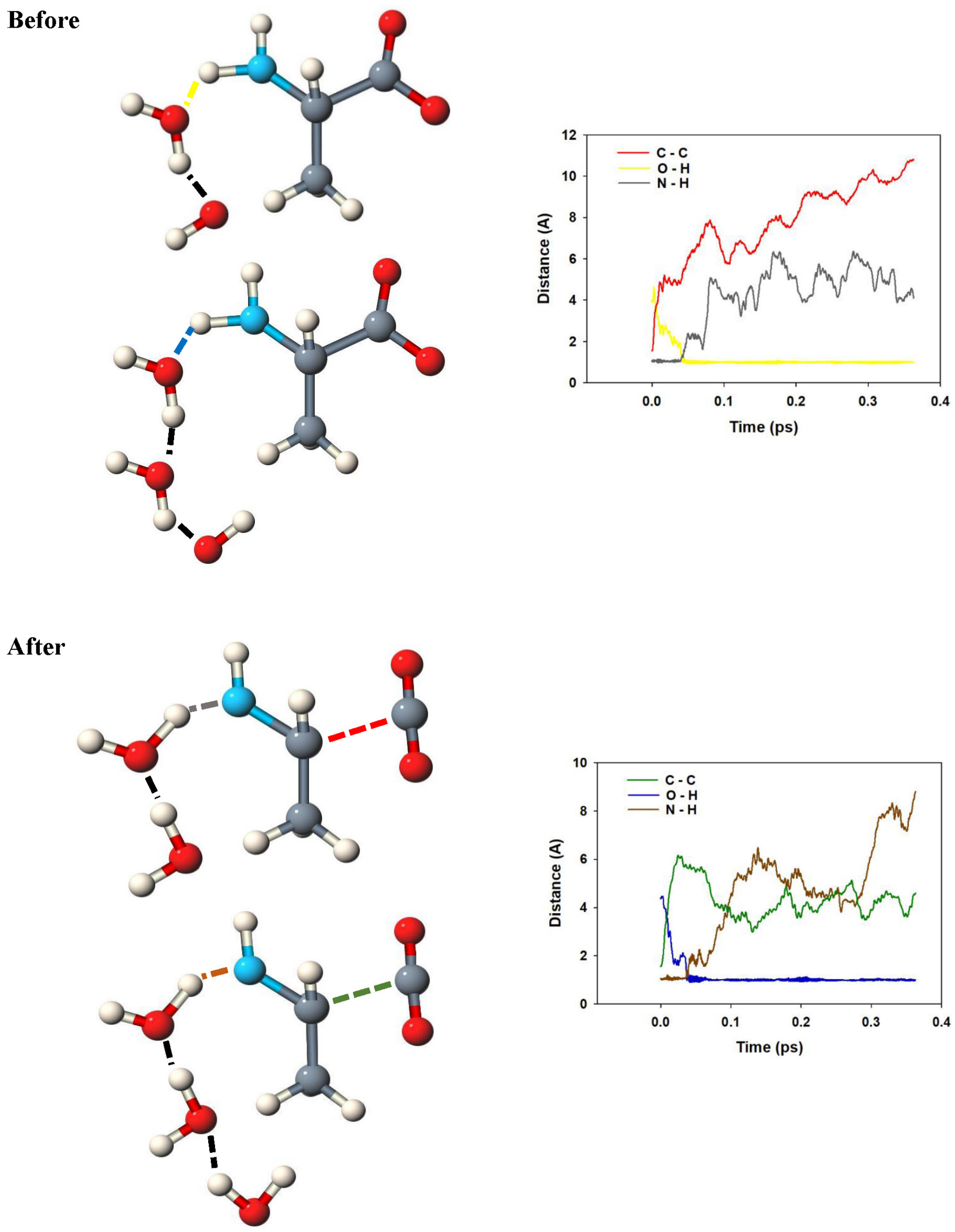

FIG. 11. One alanine molecule and one alanine radical in solution with two OH radicals. The initial temperature is 300K.

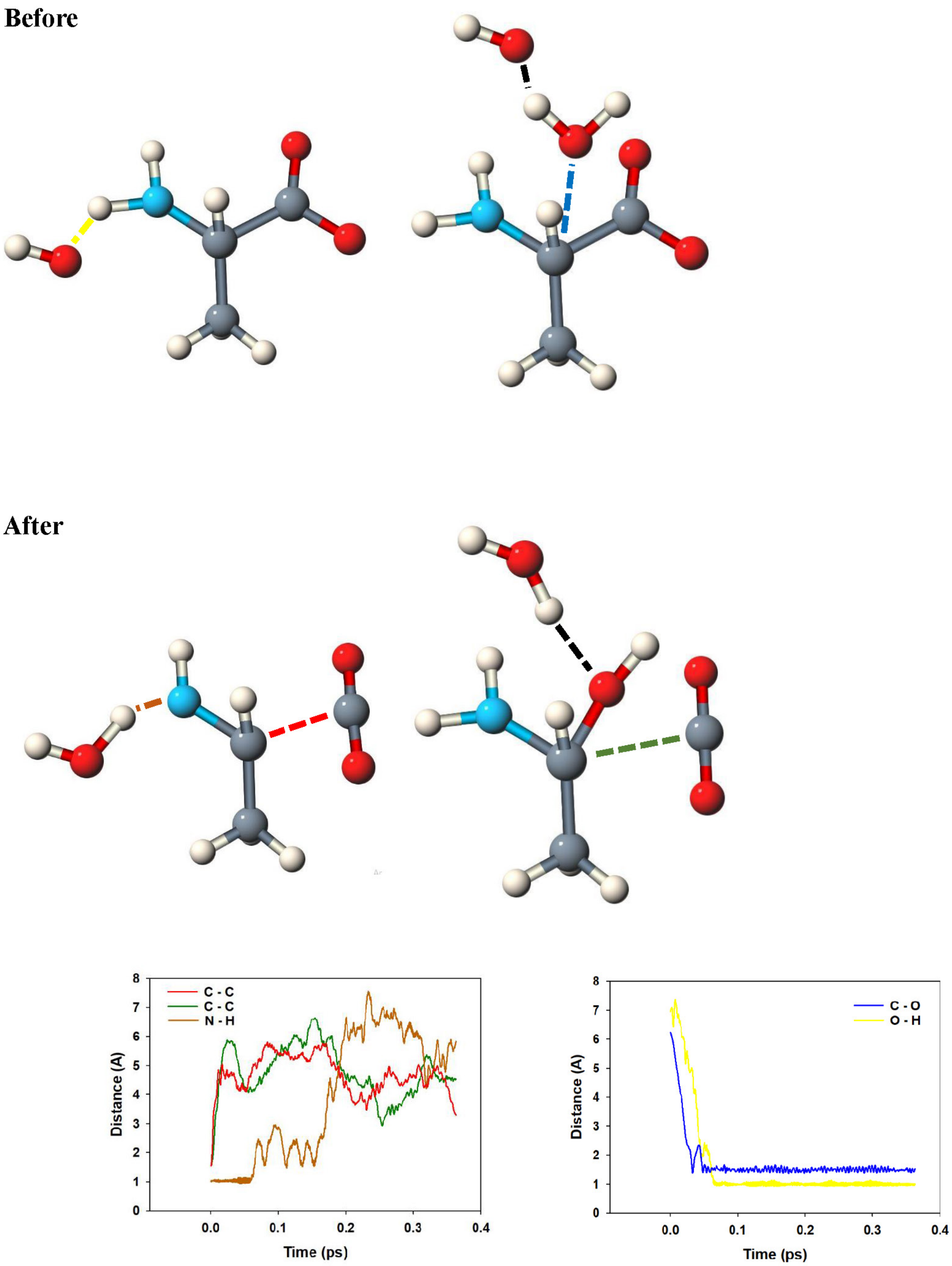

FIG. 12. One alanine and one alanine radical in solution with two OH radicals. The initial temperature is 400K.

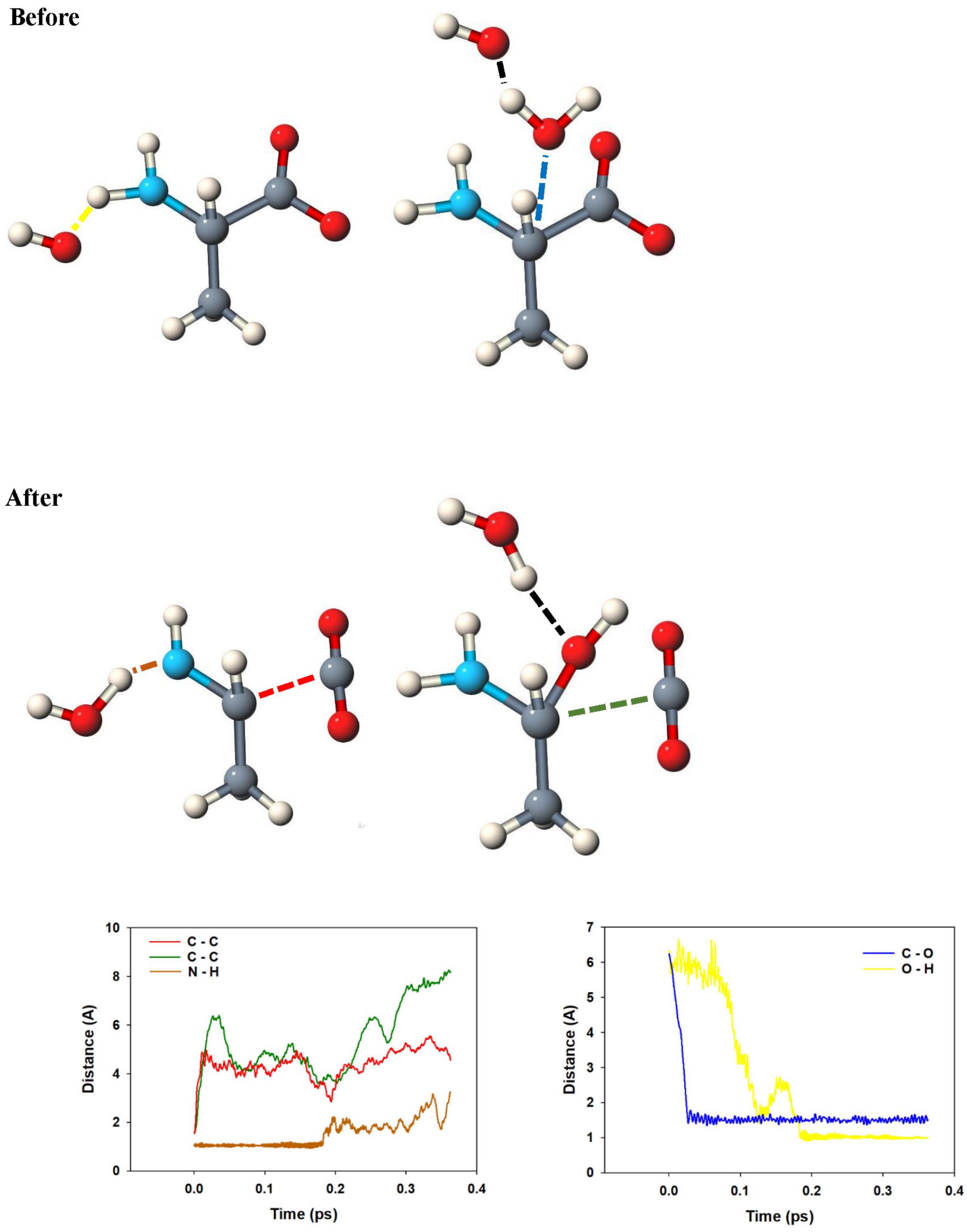

FIG. 13. One alanine and one alanine radical in solution with two OH radicals. The initial temperature is 500K.

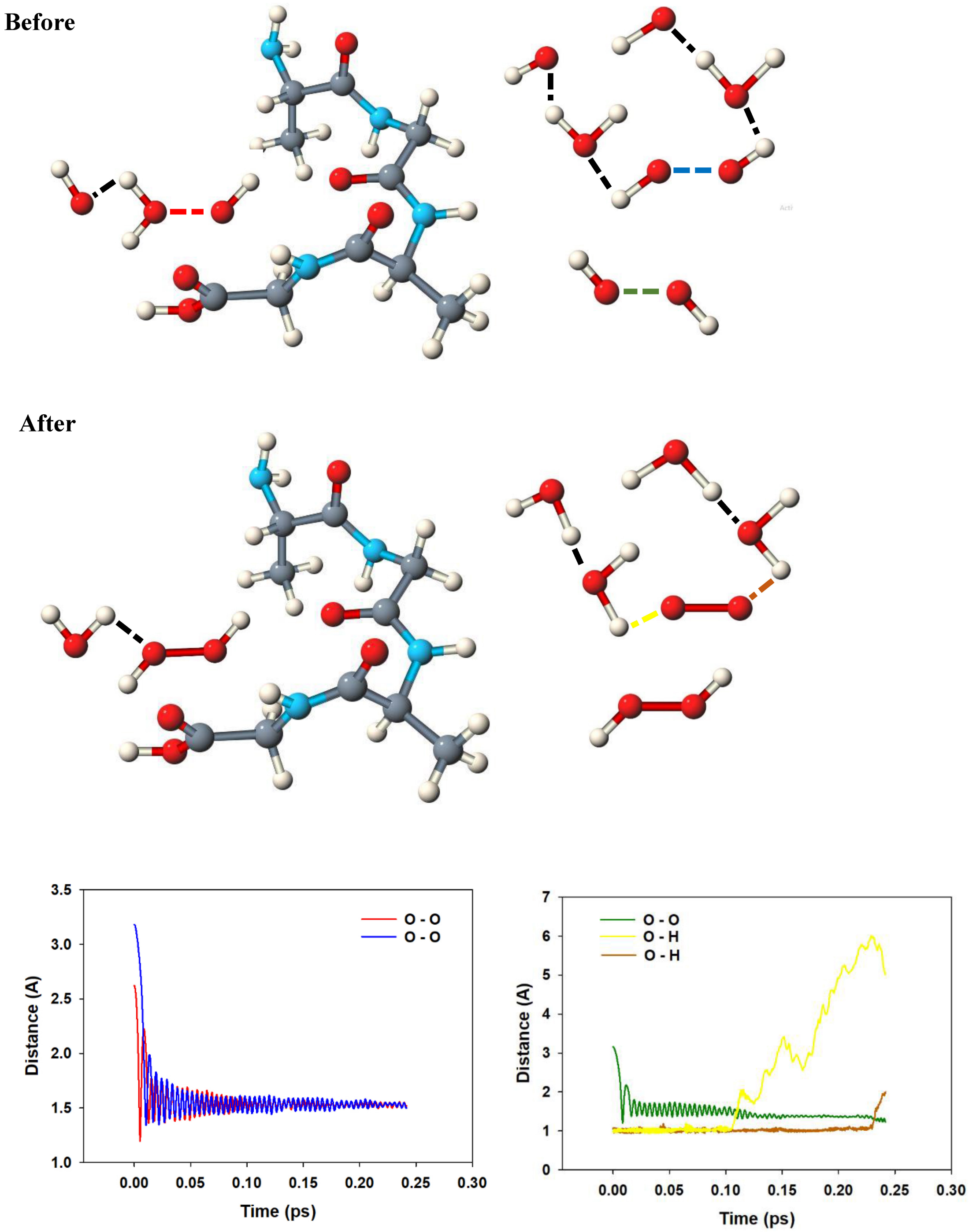

FIG. 14. Peptide in solution with eight OH radicals. The initial temperature is 300K.

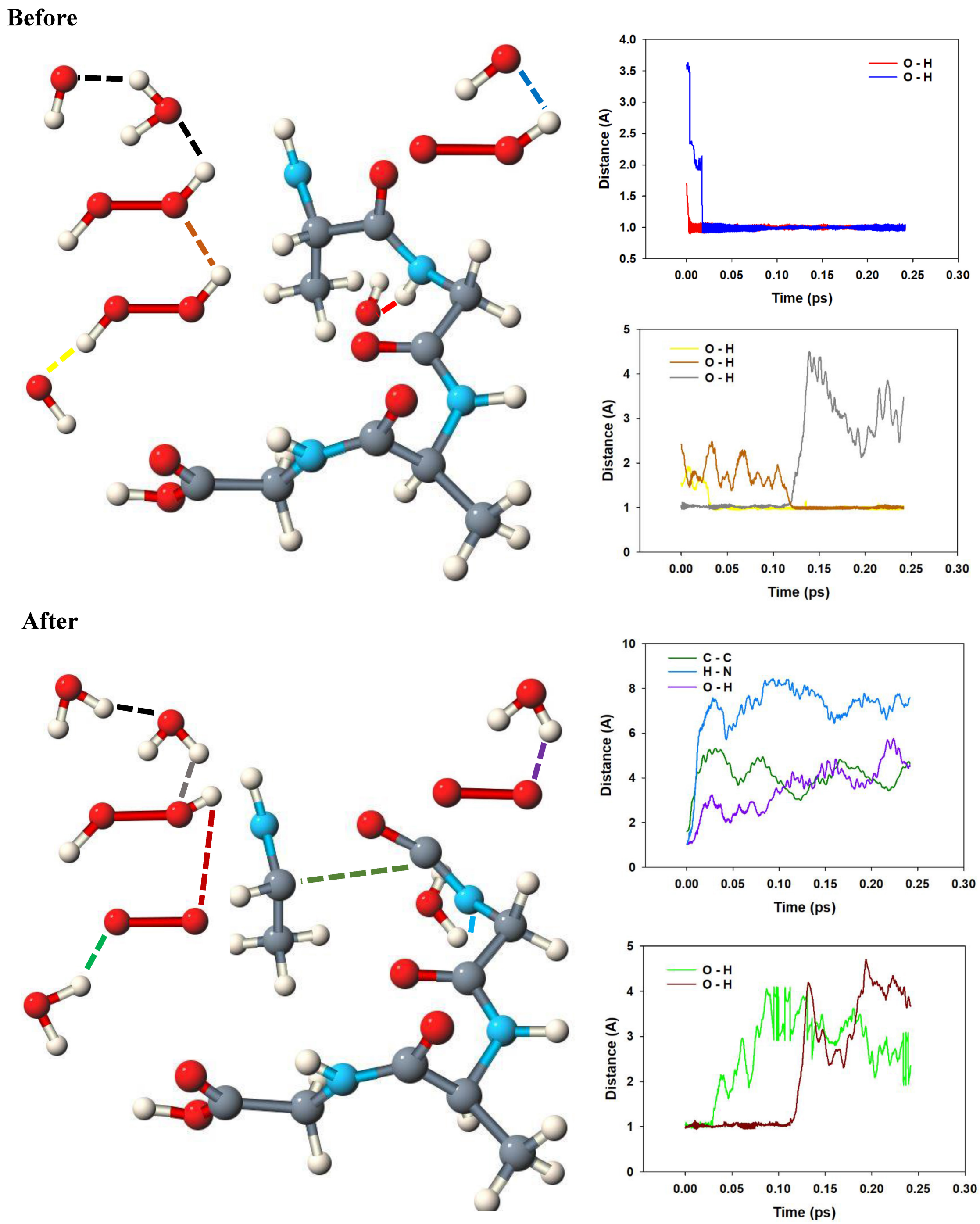

FIG. 15. Peptide in solution with twelve OH radicals. The initial temperature is 300K.

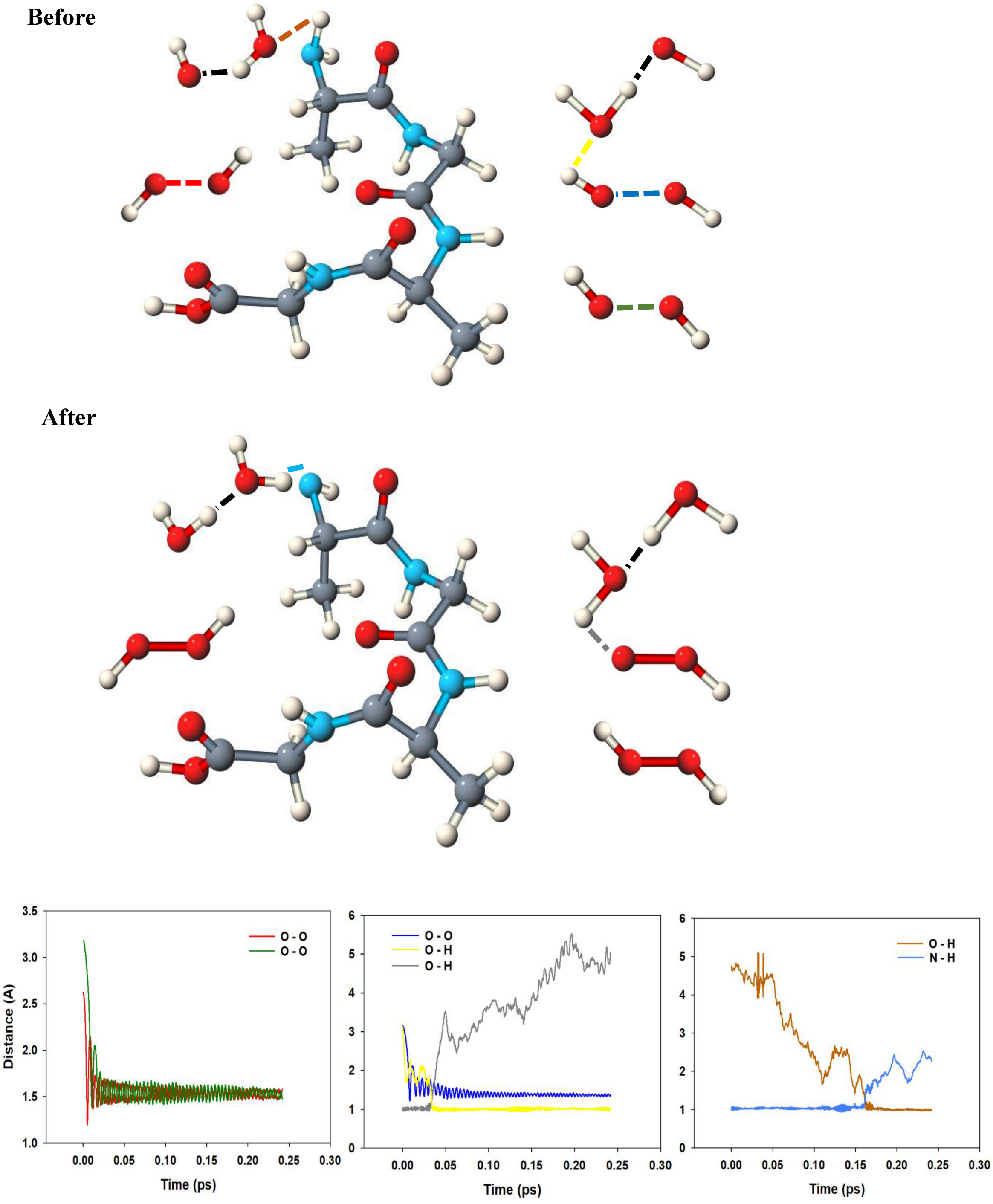

FIG. 16. Peptide in solution with eight OH radicals. The initial temperature is 400K.

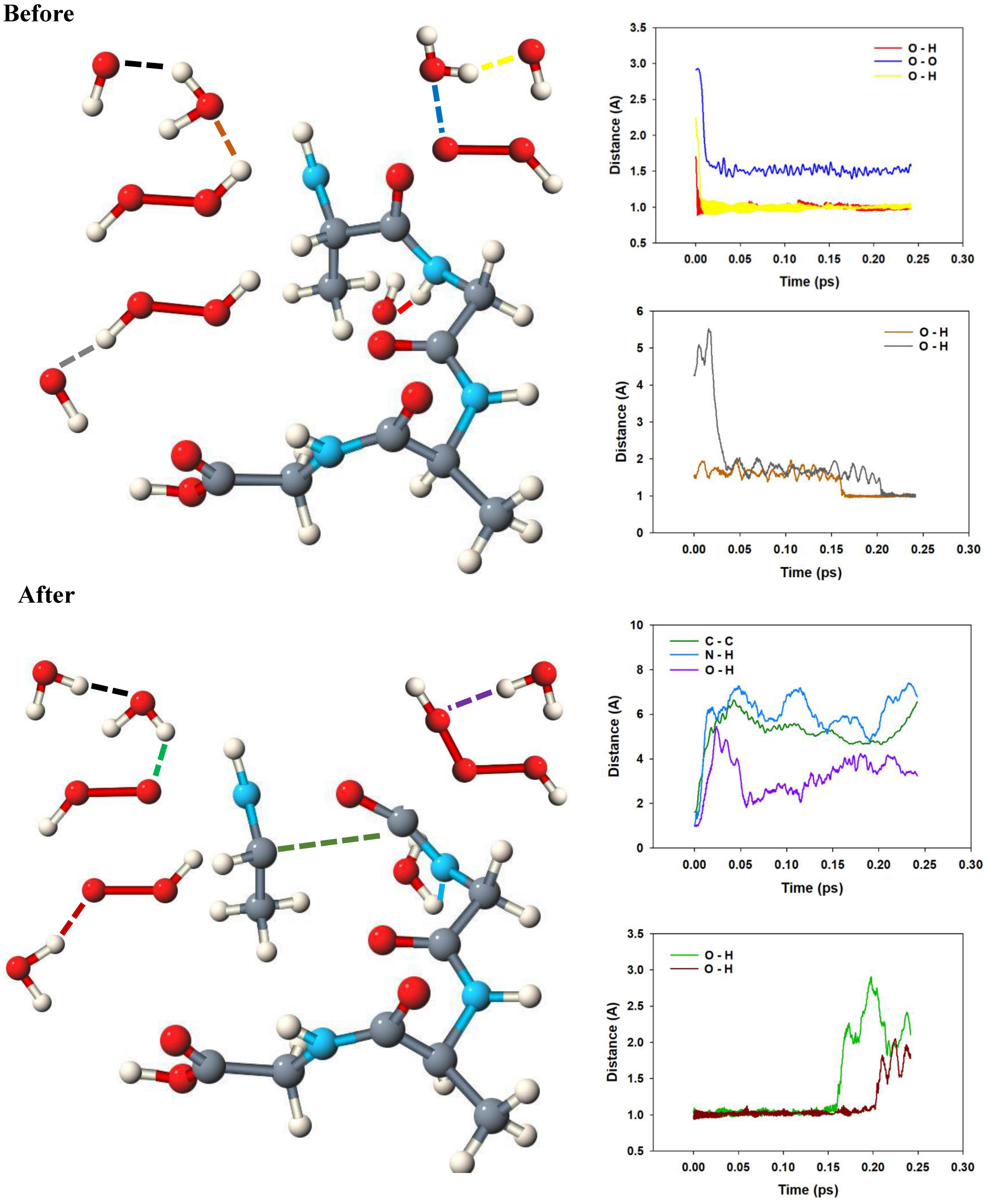

FIG. 17. Peptide in solution with twelve OH radicals. The initial temperature is 400K.

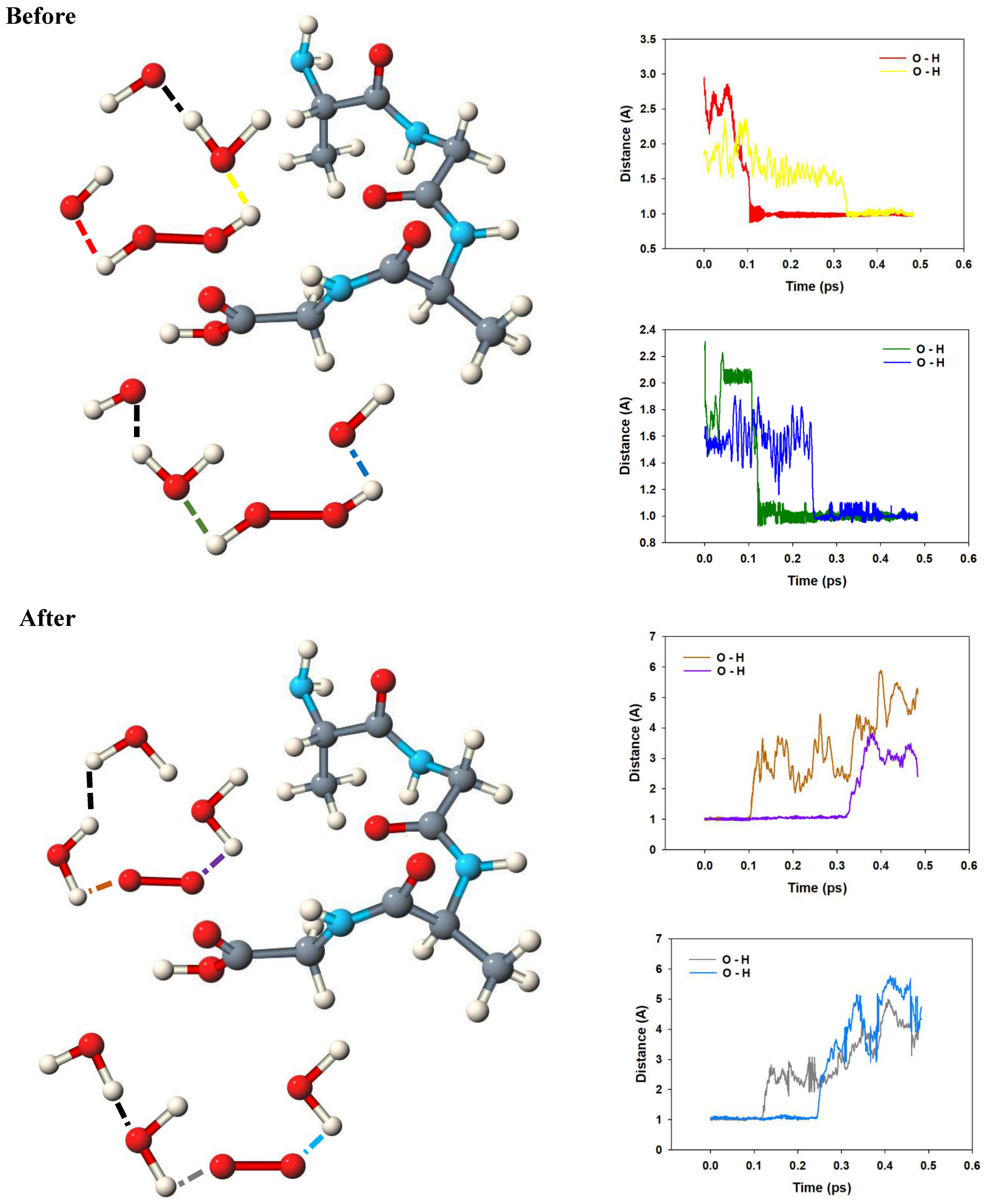

FIG. 18. Continuation of the run with the peptide in solution with twelve OH radicals. The initial temperature is 300K.

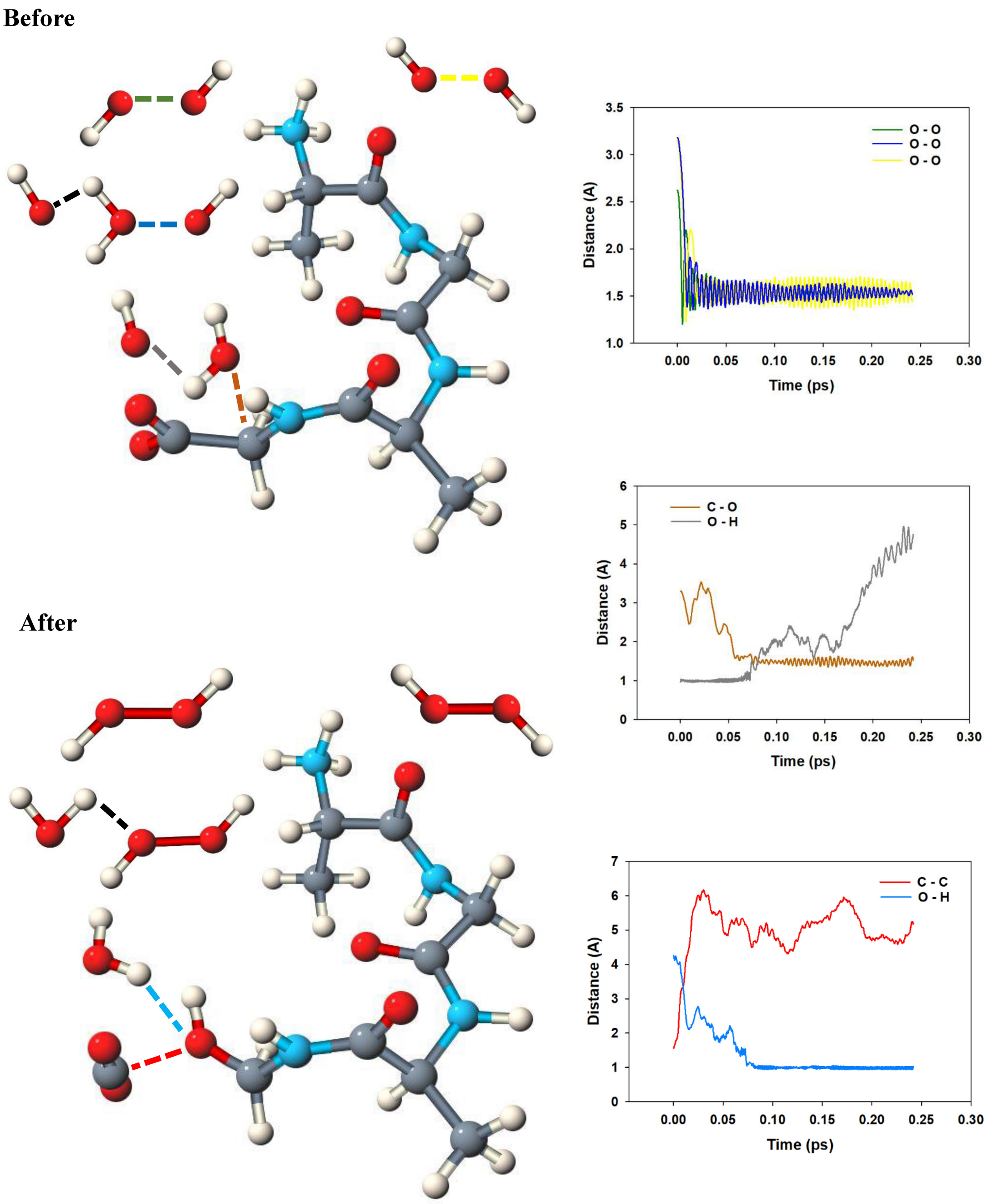

FIG. 19. Peptide zwitterion in solution with eight OH radicals. The initial temperature is 300K.

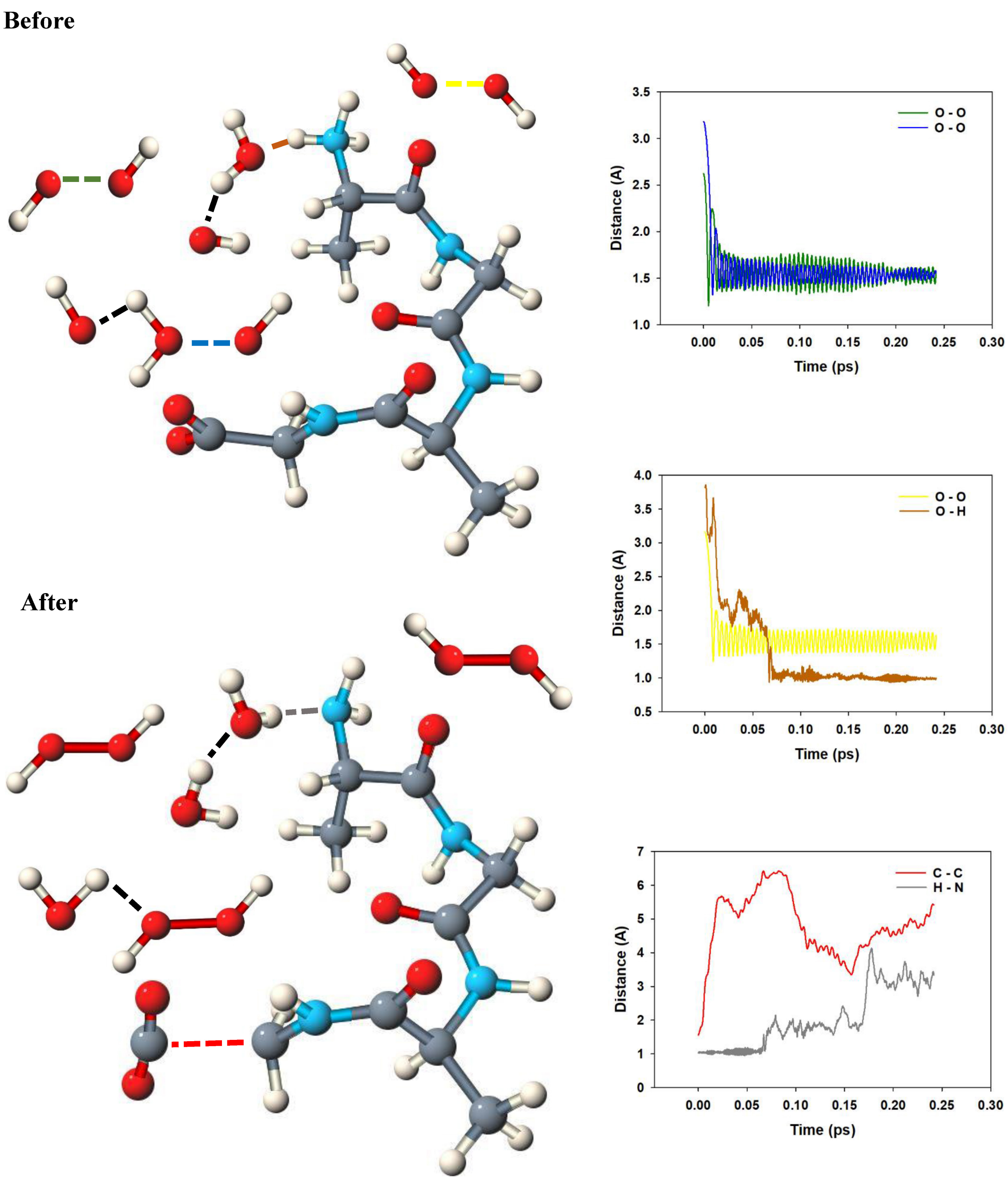

FIG. 20. Peptide zwitterion in solution with eight OH radicals. The initial temperature is 400K.
