# Supplementary figures and images for: Ab-Initio Molecular Dynamics Simulation of Condensed-Phase Reactivity: The Electrolysis of Amino Acids and Peptides
Source: Molecules. 2020 Nov 19;25(22):5415. doi: 10.3390/molecules25225415 (PMC7699423; doi:10.3390/molecules25225415)

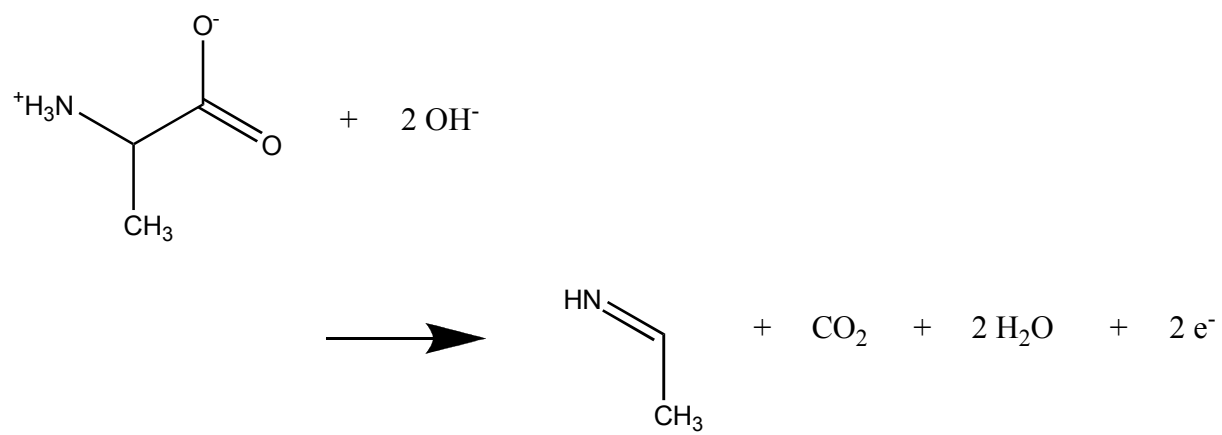

Supplement: Supplementary file 1 [file molecules-25-05415-s001.zip › frank/Fig4.pdf]

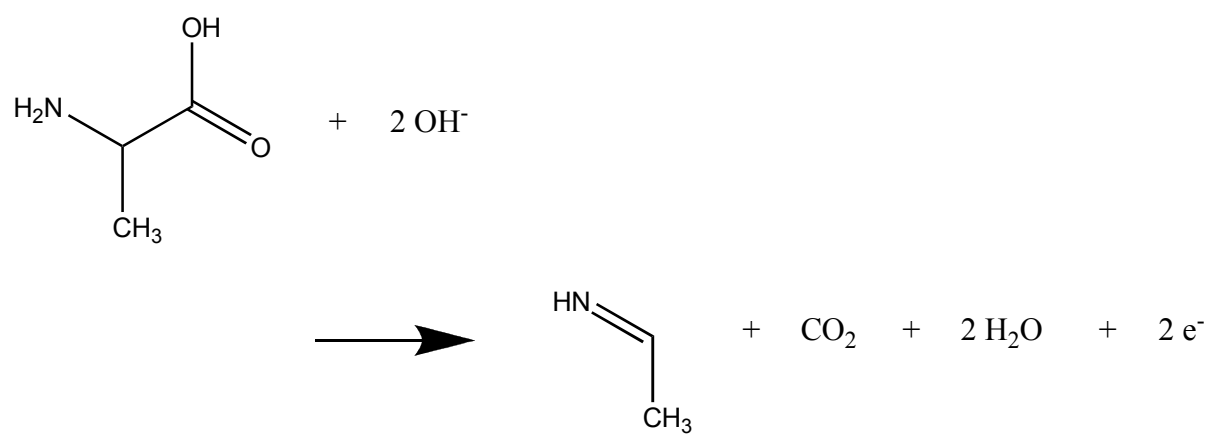

Supplement: Supplementary file 1 [file molecules-25-05415-s001.zip › frank/Fig5.pdf]

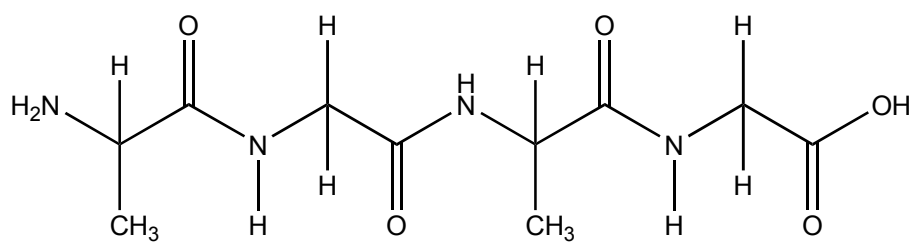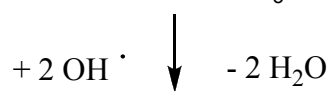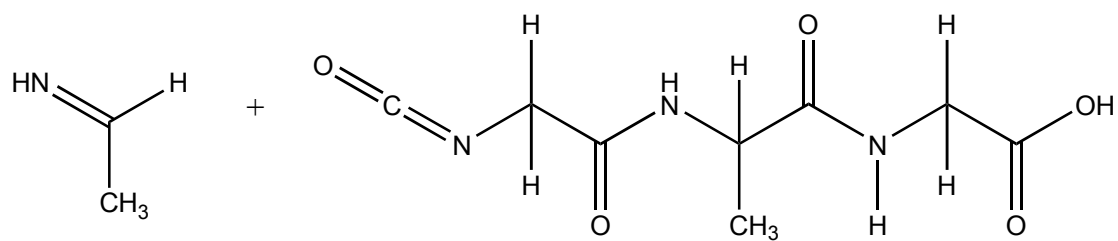

Supplement: Supplementary file 1 [file molecules-25-05415-s001.zip › frank/Fig7.pdf]
